# Confined Ru Sites in a 13X Zeolite for Ultrahigh H_2_ Production from NH_3_ Decomposition

**DOI:** 10.1021/jacs.3c05092

**Published:** 2023-06-21

**Authors:** Kwan Chee Leung, Sungil Hong, Guangchao Li, Youdong Xing, Bryan Kit Yue Ng, Ping-Luen Ho, Dongpei Ye, Pu Zhao, Ephraem Tan, Olga Safonova, Tai-Sing Wu, Molly Meng-Jung Li, Giannis Mpourmpakis, Shik Chi Edman Tsang

**Affiliations:** † Wolfson Catalysis Centre, Department of Chemistry, University of Oxford, Oxford OX1 3QR, U.K.; ‡ Department of Chemical Engineering, University of Pittsburgh, Pittsburgh, Pennsylvania 15261, United States; § Department of Applied Physics, Hong Kong Polytechnic University, Hong Kong, P. R. China; ∥ Paul Scherrer Institut, WLGA/217, Forschungsstrasse 111, 5232 Villigen PSI, Switzerland; ⊥ National Synchrotron Radiation Research Center, Hsinchu 30076, Taiwan

## Abstract

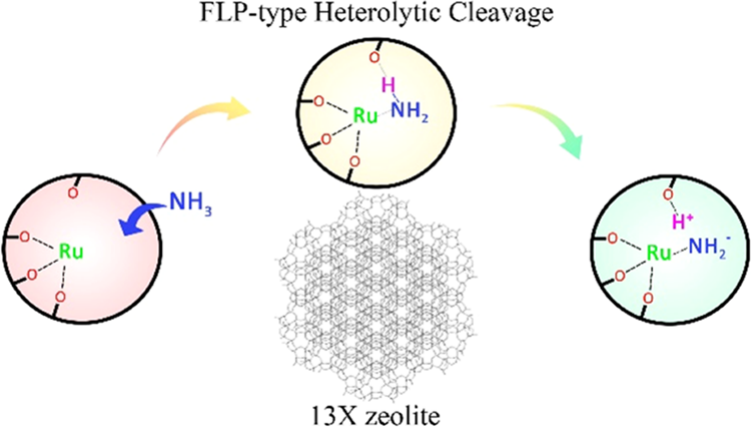

Catalytic NH_3_ synthesis and decomposition offer a new promising way to store and transport renewable energy in the form of NH_3_ from remote or offshore sites to industrial plants. To use NH_3_ as a hydrogen carrier, it is important to understand the catalytic functionality of NH_3_ decomposition reactions at an atomic level. Here, we report for the first time that Ru species confined in a 13X zeolite cavity display the highest specific catalytic activity of over 4000 h^–1^ for the NH_3_ decomposition with a lower activation barrier, compared to most reported catalytic materials in the literature. Mechanistic and modeling studies clearly indicate that the N–H bond of NH_3_ is ruptured heterolytically by the frustrated Lewis pair of Ru^δ+^–O^δ−^ in the zeolite identified by synchrotron X-rays and neutron powder diffraction with Rietveld refinement as well as other characterization techniques including solid-state nuclear magnetic resonance spectroscopy, in situ diffuse reflectance infrared transform spectroscopy, and temperature-programmed analysis. This contrasts with the homolytic cleavage of N–H displayed by metal nanoparticles. Our work reveals the unprecedented unique behavior of cooperative frustrated Lewis pairs created by the metal species on the internal zeolite surface, resulting in a dynamic hydrogen shuttling from NH_3_ to regenerate framework Brønsted acid sites that eventually are converted to molecular hydrogen.

## Introduction

Sustainable and carbon-free energy technologies must be extensively developed in order to bring a solution to the increasing energy and environmental issues nowadays. Green hydrogen has been indicated as one of the promising energy carriers for storing the electricity generated from renewable energy sources.^1^ However, inconvenient transportation of hydrogen and safety concerns limit its applications. In recent years, there has been an increasing interest in the strategy that stores hydrogen in the form of stable chemicals, which can be decomposed back to hydrogen when required.^2^ Ammonia (NH_3_) is one of the potential chemicals that can fulfill the requirements. The synthesis of green ammonia from renewables and its storage and transportation have already been well-developed recently.^3^ Additionally, the generation of hydrogen from ammonia decomposition only produces nitrogen as a side product, which is totally carbon-free and environmentally friendly. Therefore, designing high-performance catalysts for ammonia decomposition is important for achieving sustainable development of hydrogen energy.

Porous materials such as zeolites and metal–organic frameworks (MOFs) have received increasing attention based on their widespread applications in different fields, with the advantages of being low cost and environmentally friendly. One of the interesting applications of porous materials is that they can act as support to stabilize isolated metal sites for industrial catalysis, energy storage, and conversion. Zeolites contain specific pores, cages, and adsorption sites that allow confined formation and stabilization of single metal sites. The well-defined structure and controllable acidity of zeolites make the metal-loaded zeolites promising catalysts for small-molecule chemistry, including carbon dioxide hydrogenation,^4^ methane to methanol conversion,^5−7^ and ammonia decomposition,^8−11^ to name but a few. In addition, “confinement effects” in their molecular cavities make zeolites behave as solid “polar” solvents for heterogeneous dissociation and some consequences of it can be treated in analogy to solvent effects. The description of zeolites as solid solvents was first introduced in 1979 by Barthomeuf who pointed out the effect of the electric field gradient (due to the aluminum and cation distribution) on the alkane reactivity.^12^ According to this, under the influence of such field gradients, molecules can be polarized to different extents depending on the cage type, with the appearance of induced dipoles and multipoles, weakening or strengthening their bond energy levels.^13^ Besides, by controlling the polarization electric field, nitrogen activation can be promoted over single-atom catalysts.^14^ It would therefore be interesting to see how the metal-ion-doped zeolite catalysts can activate ammonia to N_2_/H_2_.

The classical mechanism of catalytic ammonia decomposition over metal nanoparticles (NPs) consists of stepwise homolytic cleavages of N–H bonds and recombination of N and H as NH_3_ → 1/2N_2_ + 3/2H_2_ with Δ*H* = +46 kJ mol^–1^, where N–H bond cleavage is generally thought to be rate-determining.^8^ Interestingly, the frustrated Lewis pair (FLP) in homogeneous catalytic systems represent another way to activate N–H in amines or related polar molecules in a stepwise heterolytic manner. Stephan et al.^15^ defined that FLP in homogeneous systems is a pair of Lewis acid and Lewis base sterically hindered and not able to form a classic Lewis acid–base dative adduct. The spatial and coordination frustration gives high intrinsic catalytic activity for the Lewis pair to activate small molecules such as ammine in a polar solvent.^15,16^ Ma et al.^17^ further elaborated the concept of FLP into other systems, which included combinations of free Lewis acids and bases via equilibria in catalytic systems as FLP. FLPs in materials for heterogeneous catalysis such as zeolites,^18−20^ metal–organic frameworks,^21^ two-dimensional (2D) materials,^22^ polymers,^23^ and CeO_2_,^24−26^ have also been recently reported.

In this work, we report for the first time that ammonia decomposition ona Ru-loaded zeolite involves the heterolytic cleavage of ammonia via an FLP-type mechanism, which is different from the homolytic pathway over metal NPs with a lower activation barrier. The Ru single sites in the 13X zeolite also show a more superior catalytic performance for ammonia decomposition, compared with the catalysts in the literature, due to their atomic dispersity and strong confinement effects in a high dielectric environment. This work could open up a new class of catalysts for the heterogeneous activation of ammonia energy and environmentally friendly applications.

## Results and Discussion

### Catalytic Performance of Ammonia Decomposition

**Figure 1:**
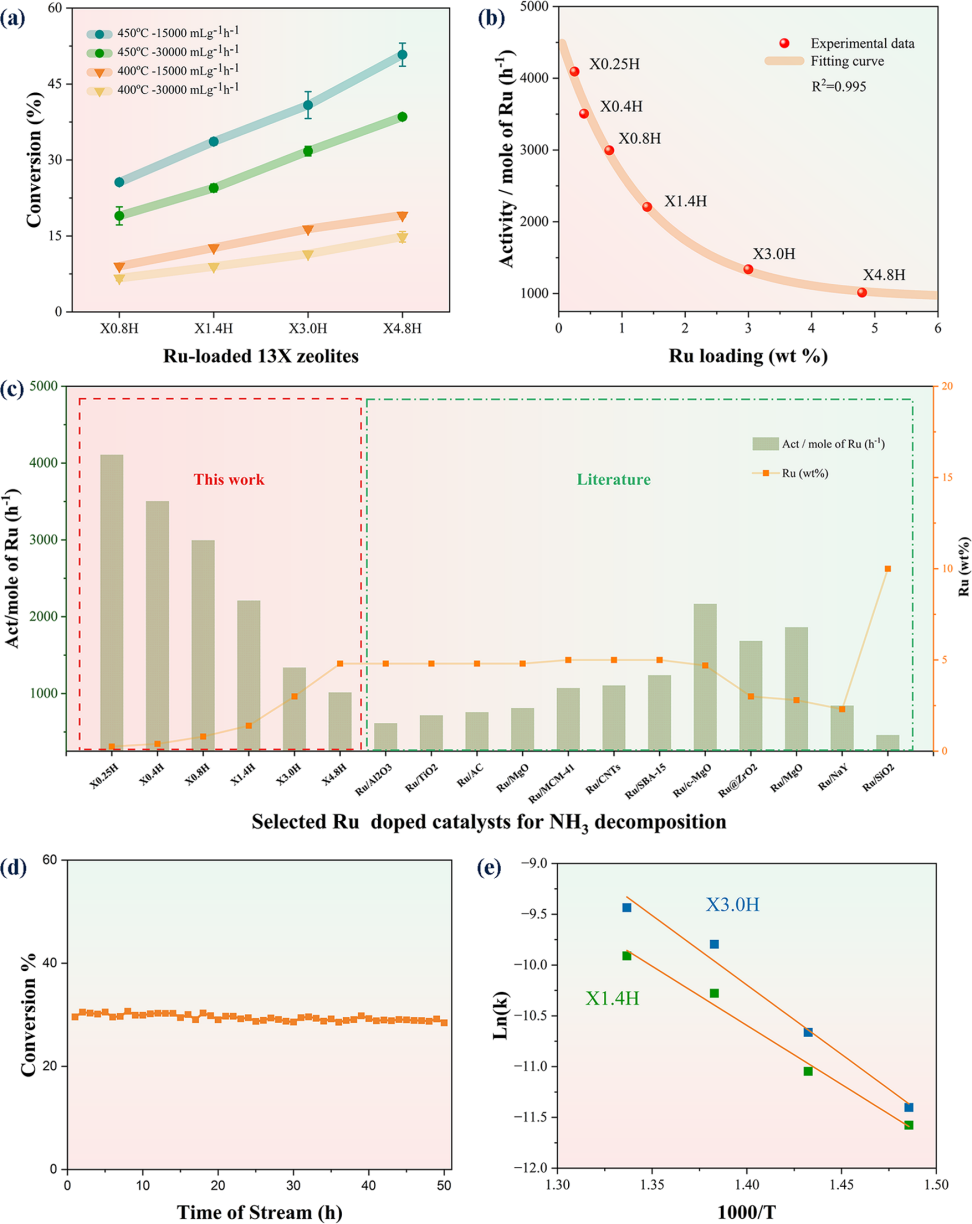
(a) NH_3_ conversion proportion of the Ru-loaded Na13X zeolites under four different conditions: 450 °C with 15 000 mL g_cat_^–1^ h^–1^, 450 °C with 30 000 mL g_cat_^–1^ h^–1^, 400 °C with 15 000 mL g_cat_^–1^ h^–1^, and 400 °C with 30 000 mL g_cat_^–1^ h^–1^. (b) Trend of the activity per mole of Ru with different Ru loadings of Ru-loaded Na13X zeolites at 450 °C with 30 000 mL g_cat_^–1^ h^–1^ for NH_3_ decomposition. (c) Catalytic performance of Ru-loaded Na13X zeolites compared with the literature data at 450 °C with 30 000 mL g_cat_^–1^ h^–1^. All of the Ru loadings from this work were obtained by inductively coupled plasma mass spectrometry (ICP-MS) (Table S6). (d) No apparent change in conversion (%) during the stability test of X1.4H for 50 h. (e) Arrhenius plots of X1.4H and X3.0H.

Catalytic NH_3_ decomposition to N_2_ and H_2_ over our Ru-loaded zeolite samples was first tested in the following pure NH_3_ gas flow conditions at s.t.p: (i) 30 000 mL g_cat_^–1^ h^–1^ at 400 °C, (ii) 15 000 mL g_cat_^–1^ h^–1^ at 400 °C, (iii) 30 000 mL g_cat_^–1^ h^–1^ at 450 °C, and (iv) 15 000 mL g_cat_^–1^ h^–1^ at 450 °C. The conversion of the prereduced Ru-loaded 13X zeolites at different contents of Ru precursors (X0.8H, X1.4H, X3.0H and X4.8H, where the number indicates the wt % Ru loading of the samples and **H** stands for sample after H_2_ treatment) is shown in Figure 1a. It can be seen that the conversion rate progressively increases when the Ru loading increases from 19.0% (X0.8H) to 38.5% (X4.8H) for 30 000 mL g_cat_^–1^ h^–1^ at 450 °C. It is also interesting to note from Figure 1b that the specific activity (i.e., mole of NH_3_ converted per mole of Ru loaded) is higher when lower Ru content is used, typically from 1013.9 h^–1^ (X4.8H) to 2994.2 h^–1^ (X0.8H). The specific activity levels off at 1000 h^–1^ when the Ru loading approaches ∼5 wt %. This is likely accounted for by the fact that smaller or evenly isolated Ru sites are more active than corresponding larger NPs on the zeolite samples. Two more samples with lower Ru loading were therefore synthesized based on that and named X0.25H and X0.4H (0.25 and 0.4 wt% Ru loadings, respectively). The two low Ru-loaded samples fit the trendline and provide ultrahigh specific activities in which X0.25H provides over 4000 h^–1^ specific activity (4108.5 h^–1^ for X0.25H and 3502.7 h^–1^ for X0.4H). Figure 1c shows the catalytic performance of our catalysts as compared to some selected catalysts from the literature with 30 000 mL g_cat_^–1^ h^–1^ at 450 °C. A more extensive comparison with some recently reported Ru-based catalysts in the literature can be found in Table 1. As seen from Table 1, the specific activities of our Ru-loaded 13X catalysts especially with low Ru contents out-perform most of the state-of-the-art catalysts such as Ru/carbon nanofibers (Ru/CNFs), Ru/carbon nanotubes (Ru/CNTs), and Ru/MgO. Notably, X1.4H was continuously tested in a flow reactor for a 50 h period. Figure 1d shows no significant drop in the % conversion of NH_3_ decomposition. This indicates the Ru sites in the 13X zeolite and the 13X zeolite support itself are stable throughout the catalysis. Kinetic studies of X1.4H and X3.0H have also been performed, and Arrhenius plots are obtained by the linear fitting of Ln (reaction rate) vs 1/*T* (Figure 1e). The apparent activation energy (*E*_a_) values of X1.4H (small Ru cluster) and X3.0H (Ru NPs) were determined to be +94.4 kJ mol^–1^ (i.e., +0.98 eV) and +127.1 kJ mol^–1^ (i.e., +1.32 eV), respectively. Thus, the former kinetic barrier for the ammonia decomposition is significantly lower than that of a typical value of 120–130 kJ mol^–1^ obtained from those of Ru NPs over a wide range of supports.^8−11^

**Table 1:** Catalytic Performance of Ru-Loaded 13X Zeolites Compared with the Literature Data for NH_3_ Decomposition

		Ru (wt %)	*T* (°C)	WHSV (mL g_cat_^–1^ h^–1^)	conv. (%)	mol NH_3_/mol Ru (h^–1^)	reference
Ru/13X	X0.25H	0.25	450	30 000	8.1	4108.5	[this work]
	X0.4H	0.4	450	30 000	11.1	3502.7	[this work]
	X0.8H	0.8	450	30 000	19.0	2994.2	[this work]
	X1.4H	1.4	450	30 000	24.5	2207.3	[this work]
	X3.0H	3.0	450	30 000	31.8	1337.1	[this work]
	X4.8H	4.8	450	30 000	38.5	1013.9	[this work]
Ru/CNFs		3.2	500	30 000	99.0	3908.6	(29)
Ru/CeO_2_		1.0	350	22 000	ca. 32.0	2964.7	(30)
Ru/C12A7:e^–^		2.2	450	15 000	ca. 99.9	2868.4	(8)
Ru/Cr_2_O_3_		5.0	600	30 000	99.9	2524.2	(31)
Ru/MgO		3.5	450	36 000	52.7	2282.7	(32)
Ru/c-MgO		4.7	450	30 000	80.6	2166.6	(33)
Ru/C12A7:e^–^		2.2	400	15 000	70.0	2009.9	(8)
Ru/BHA		2.7	450	60 000	ca. 20.8	1918.1	(34)
Ru/MgO		2.8	450	30 000	41.3	1863.5	(35)
Ru@ZrO_2_		3.0	450	30 000	40.0	1684.5	(36)
Ru/MgO-MIL		3.1	450	15 000	ca. 70.0	1426.4	(37)
Ru/La_0.33_Ce_0.67_		1.8	450	6000	100.0	1403.8	(38)
Ru/SBA-15		5.0	450	30 000	49	1238.1	(39)
Ru/La_2_O_3_		4.8	450	18 000	72.8	1149.7	(40)
Ru–Ba(NH_2_)_2_		4.4	400	60 000	ca. 20.0	1148.5	(41)
Ru/CNTs		5.0	450	30 000	43.7	1104.2	(42)
Ru/MCM-41		5.0	450	30 000	42.4	1071.3	(39)
Ru/NaY		2.3	450	30 000	ca. 15	842.3	(10)
Ru/MgO		4.8	450	30 000	30.8	810.7	(43)
Ru/AC		4.8	450	30 000	28.7	755.4	(43)
Ru/TiO_2_		4.8	450	30 000	27.2	715.9	(43)
Ru/Al_2_O_3_		4.8	450	30 000	23.3	613.3	(43)
Ru/SiO_2_		10.0	450	30 000	36.4	459.9	(44)
Ru–Ca(NH_2_)_2_		4.6	400	60 000	ca. 8.0	439.4	(41)
Ru–Mg(NH_2_)_2_		5.0	400	60 000	ca. 4.0	202.1	(41)

To address the kinetic isotope effects (KIE) in N–H cleavage, during the testing of a Ru-loaded 13X zeolite (X1.4H), we switched the NH_3_ gas supply to ND_3_ by a rapid switching valve to the reactor before the gas chromatography (GC) detector while all of the conditions were maintained. As a result, we can deduce the reaction rates of H-containing NH_3_ and D-containing ND_3_ as *k*_H_ and *k*_D_, respectively. By examining the *k*_H_/*k*_D_, we address KIE in N–H cleavage as the rate-limiting step between unlabeled N–H cleavage for NH_3_ and labeled N–D for ND_3_.^27^ Under the conditions of 450 °C and 30 000 mL g_cat_^–1^ h^–1^, a value of *k*_H_/*k*_D_ of 1.74(1) was obtained. This value should not be taken as a quantitative measure but rather as qualitative, as the differential flow conditions may not be rigorously ensured (NH_3_ conversion <1%) since our conversion was about 15% (still far from the depletion of substrates). However, this high value suggests the more sluggish activity for the deuterium-labeled NH_3_, indicative of the KIE effect (1.4)^28^ with the heterolytic N–H bond cleavage being the rate-determining step.

### Synchrotron X-ray Diffraction and Neutron Powder Diffraction (NPD) with Refinements

In order to gain meaningful structure–activity relationships, careful synthesis of catalyst samples and detailed material characterization were carried out, as shown in the Supporting Information (SI). Both powdered synchrotron X-ray diffraction (SXRD) and laboratory X-ray diffraction (XRD) of Ru-loaded 13X zeolite samples (Figures S1 and S2) were characterized by highly crystalline peaks. However, progressive peak shifts and peak intensity attenuations are clearly observed when higher Ru loadings are used, i.e., X3.0H and X4.8H (3.0 and 4.8 wt % Ru loadings, respectively), while there is no noticeable change for the lower Ru-content samples i.e., X0.8H and X1.4H (0.8 and 1.4 wt % Ru loadings, respectively) compared to the pristine sample. It is accepted that aggregation of metal atoms (Ru in this case) in small zeolite cavities after H_2_ pretreatment can lead to distortion of lattice parameters (peak shifts) and even phase destruction to account for peak intensity attenuation.^45,46^ Thus, the X1.4 (as-synthesized sample before H_2_ treatment) and X1.4H samples were selected for detailed characterization. Despite the fact that X0.25H shows the highest TOF per site in such extremely dilute Ru loading (isolated Ru sites), X1.4H was used as a benchmark. This is because it is rather challenging to conclude the atomic position and structure of such a low level of Ru sites (i.e., X0.25H) by the diagnostic SXRD and NPD with refinements, and also X-ray absorption spectroscopy (XAS). X1.4H is the one with the highest Ru loading from the synthesized samples and at the same time, preserving the porous structure. The partially collapsed microporous structure of the 13X zeolite at a ≥3 wt % Ru loading (see Figures S1 and S2) could hinder the accessibility of Ru sites. However, we also see the progressive decrease in the TOF upon increasing Ru loading (<3 wt %) even before the collapse of the microporosity, as shown in Figure 1b. This indicates the effect of changing nature of Ru species upon the activity.

**Figure 2:**
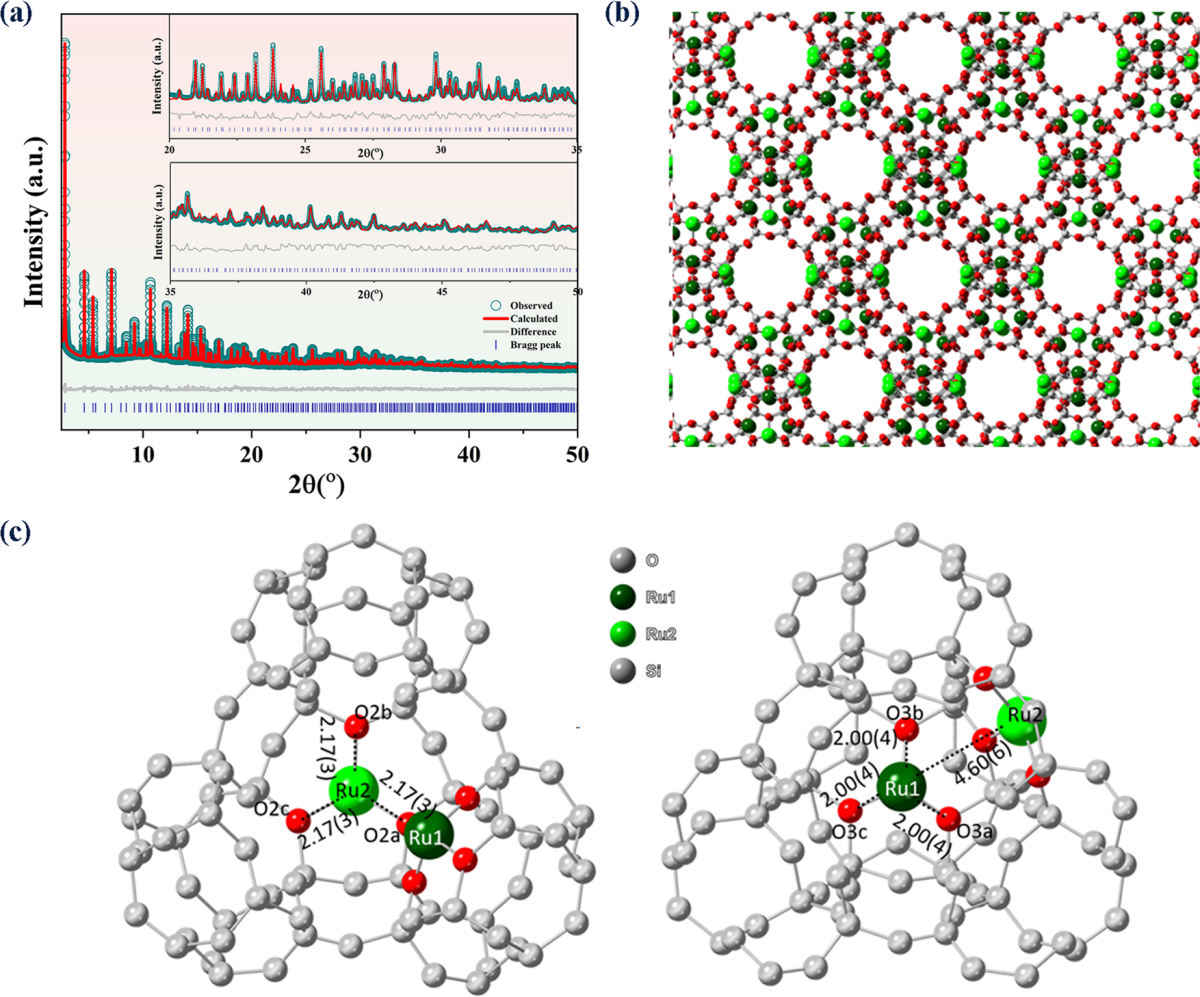
(a) Rietveld refinement profiles of SXRD data (λ = 0.708721(7) Å) of X1.4H (*R*_wp_ = 7.187%, *R*_exp_ = 0.221%, gof = 32.507). (b) Zeolite structure showing the determined Ru sites (Ru1 and Ru2) of X1.4H via SXRD-Rietveld refinement. The atoms are labeled according to element (dark green = Ru1, light green = Ru2, red = O, and gray = Si and Al). (c) Zeolite structures with the determined Ru sites (Ru1 and Ru2) of X1.4H with the bond length information (in Å) retrieved via SXRD-Rietveld refinement. All O atoms turned into gray color except the specific O atoms that are connected to Ru1 and Ru2 sites.

First of all, the extended X-ray absorption fine structure (EXAFS) of the X1.4 sample (Figure S13 and Table S2) showed an isolated first sphere of six O-coordinated Ru, where [O] atoms are thought to come from the wall of 13X (substituting Na^+^ position) as well as H_2_O ligands (no Ru–Cl). Also, a longer Ru–Ru distance of 3.12(3) Å than metal bonding (2.67(4) Å) in the confined space can be observed. Such isolated Ru local coordination environments generally agree with the refined SXRD atomic model (Figure 2b,c). Rietveld refinement of the two diffractograms was performed accordingly. The high-resolution SXRD and neutron powder diffraction (NPD) enable the determination of locations of Ru metal atoms and corresponding small adsorbates that adopt the Bragg units in zeolites such as ammonia, acetone, and methanol.^19,47−49^

The nature of the Ru sites in pores of 13X can be deduced via constructing the crystal structure of the Ru-loaded 13X zeolite based upon the Rietveld refinement on high-resolution SXRD data^50^ using TOPAS-Academic 6 (Figure 2a). Particularly, the generated electron density map from the Na13X model^51^ was compared with that of X1.4H, which resulted in a Fourier difference map, i.e., the three-dimensional electron density difference map. The location of Ru sites can hence be visualized, and Ru atoms were then added to the 13X zeolite model guided by the improved fitting parameters. The refined structure of the X1.4H sample with good acceptable refinement parameters within experimental errors (Tables S7 and S8) is shown in Figure 2b,c: there were two major types of Ru sites (named Ru1 and Ru2), both of which are connected with three O sites. Ru1 sites are located on the hexagonal prisms, which are joined with the sodalite cages, while Ru2 sites are located on the six-membered rings of the unjoined hexagonal faces. There are four different types of O sites in 13X (named O1, O2, O3, and O4), and only two types of them are found in close proximity to the Ru sites, namely Ru1–O3 and Ru2–O2 with bonding distances of 2.00(4) and 2.17(3) Å, respectively, in X1.4H. The two O sites are attributed to proton-depleted Brønsted acid sites (BAS), where Ru1 and Ru2 ions replace the Na ions during the ion exchange.

**Figure 3:**
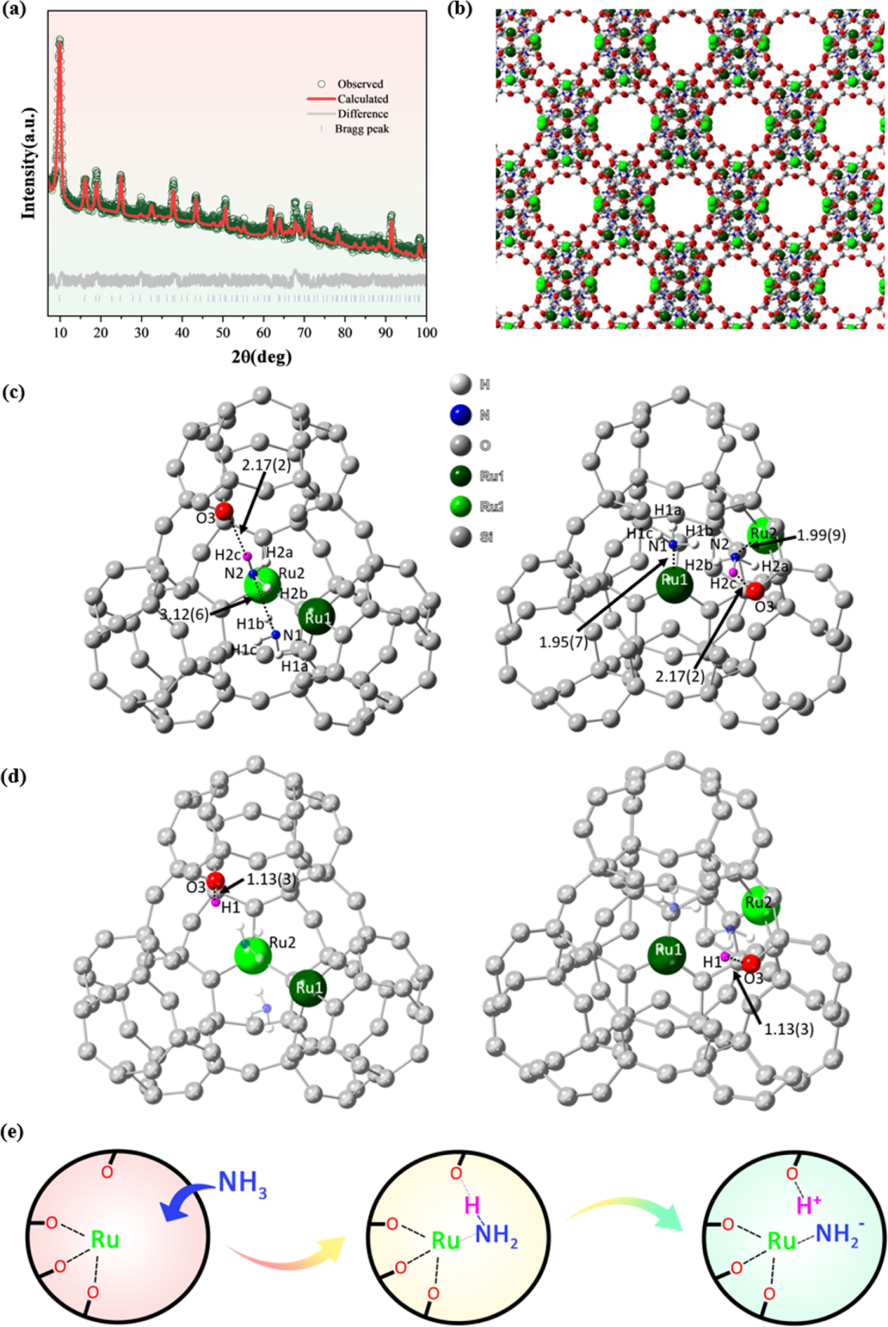
(a) Original and Rietveld refinement fit of NPD data of X1.4H-RT (*R*_wp_ = 2.375%, *R*_exp_ = 1.865%, gof = 1.2736). (b) Zeolite structure showing the determined proton sites and NH_3_ molecules of X1.4H-RT via NPD-Rietveld refinement. The atoms are labeled according to the element (dark green = Ru1, light green = Ru2, red = O, gray = Si and Al, blue = N and white = H). (c) Zeolite structure with the determined NH_3_ molecules of X1.4H-RT with the bond length information (in Å) via NPD-Rietveld refinement. All O atoms are turned into gray color except the O atom (O3) that is located close to the proton H2c (in pink) of the adsorbed NH_3_ of Ru2 and is able to undergo the FLP-type dissociation mechanism. (d) Zeolite structure containing regenerated proton sites on O atoms and NH_3_ molecules of X1.4H-450 with given bond length information (in Å) via NPD-Rietveld refinement. All O atoms are turned into gray color except the O atom (O3) that undergoes the FLP-type dissociation mechanism and the corresponding proton H1 (in pink). (e) Proposed FLP-type mechanism for the N–H cleavage that occurred between NH_3_ gas and the Ru sites in the Ru-loaded 13X zeolite.

Complementary to the SXRD data, the same crystal structure was obtained by the NPD refinement (Table S9), which is also agreeable with the optimized structure from computational simulation (see later section and SI). In addition, the nature of proton sites and the location of adsorbates (NH_3_) in Ru-loaded 13X zeolites can be refined via the use of the Rietveld refinement method in TOPAS-Academic 6, based on the high-resolution NPD data (Figures 3a,b and S24). The generated X1.4H model was compared with the neutron density maps of X1.4H with (1) NH_3_ adsorption at room temperature (X1.4H-RT) and (2) NH_3_ decomposition at 450 °C (X1.4H-450), resulting in a Fourier difference map, i.e., the three-dimensional neutron density difference map. The location of proton sites can hence be visualized, and H atoms were added to the Ru-loaded 13X zeolite models. From the NPD-Rietveld refinement of X1.4H-RT (Figure 3c and Table S10), it is interesting to identify a partially occupied proton site (site H1) binding on the framework O3 associated with adsorbed ammonia (near the Ru1 site), where no proton site density with framework O2 (near the Ru2 site) is observed. The X1.4H-RT sample clearly shows that some NH_3_ molecules are physically adsorbed around both Ru1 and Ru2 sites. In contrast, the NPD-Rietveld refinement of X1.4H-450 after the reaction with ammonia (Figure 3d and Table S11) clearly reveals that the isolated site H1 on O3 is doubled in its site occupancy value (from 0.1944(3) to 0.3959(0)). Additionally, the proton site (site H2) with framework O2 in remote from N species also becomes apparent (site occupancies from 0 to 0.3557(8)). This thus confirms for the first time that NH_3_ molecules can be dissociated into H and NH_2_ species over isolated metal sites in the zeolite at elevated temperature (Ru acting as Lewis acid), while the H atoms clearly sit on the nearby framework O atoms (O as Lewis base) to regenerate the BAS (i.e., framework protons). This activation indicates that the heterolytic cleavage of N–H bonds occurs as an FLP-type mechanism over the Ru-loaded zeolite (Figure 3e), similar to homogeneous systems^15^ where steric restrictions impose barriers for the dative bonding between the Lewis acid (metal site) and Lewis base (depleted BAS). It is also anticipated that small-molecule activation such as NH_3_ in a confined but highly polarized environment (high dielectric constant) in the zeolite cavity is akin to the favorable polar solvency used in the homogeneous systems.^52^ Interestingly, the two ammine moieties in shorter distances than adsorbed ammonia on Ru1 and Ru2 appear to be close to each other (facilitating the formation of dinitrogen species) and may eventually dehydrogenate to N_2_.

### Further Characterizations

**Figure 4:**
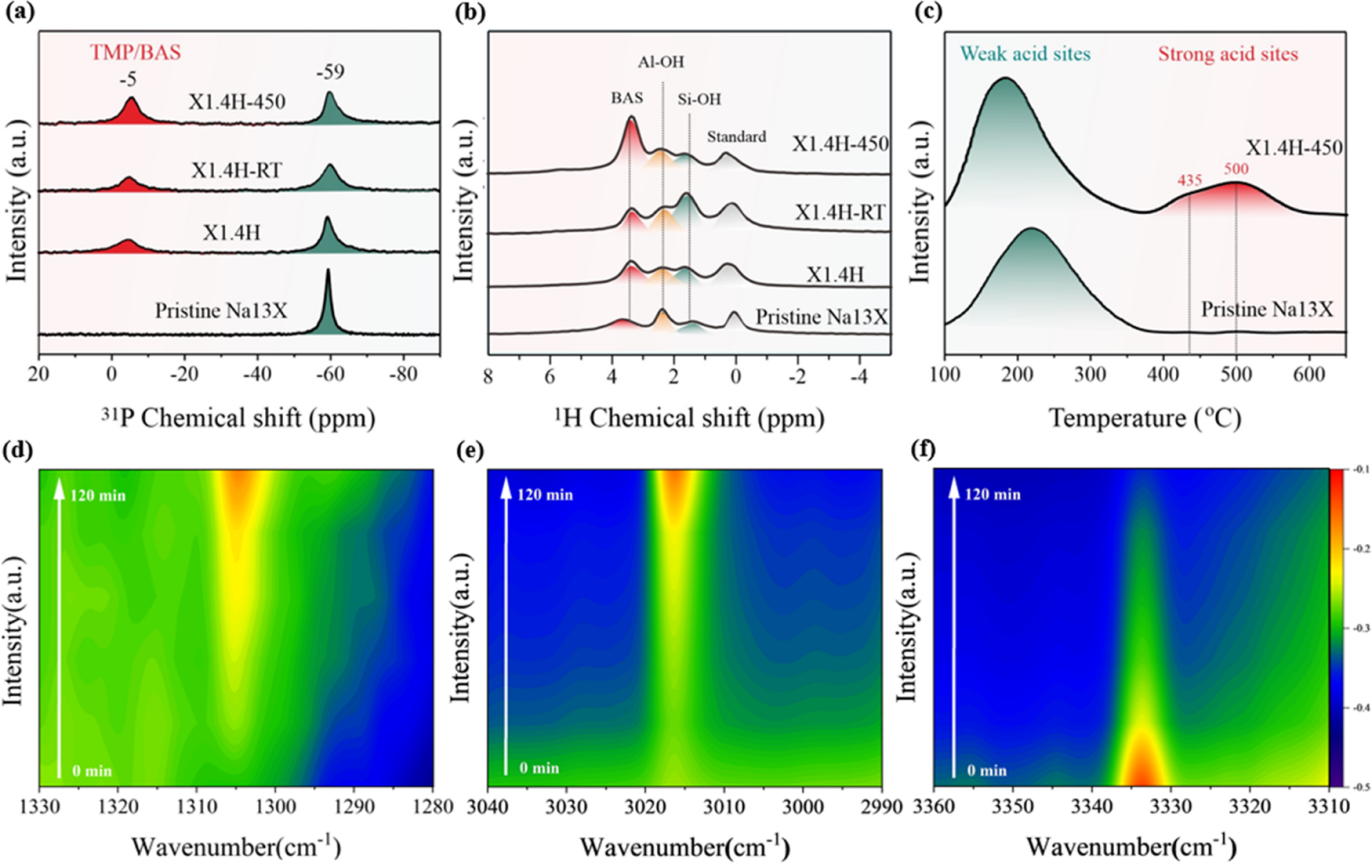
(a) ^31^P MAS NMR spectra of TMP adsorbed samples. (b) ^1^H MAS NMR spectra of the samples. (c) NH_3_-temperature-programmed desorption (TPD) of pristine Na13X and the X1.4H-450 sample with deconvolution peaks. (d–f) Changes in intensity of peaks at 1305, 3017, and 3333 cm^–1^ of in situ diffuse reflectance infrared Fourier transform spectroscopy (DRIFTS) experiments with NH_3_ adsorption of X1.4H from 0 to 120 min.

Magic angle spinning (MAS) solid-state nuclear magnetic resonance (SSNMR) with and without a probe molecule was also used to investigate proton sites in our samples before and after NH_3_ treatments. First, the samples (pristine Na13X, X1.4H, X1.4H-RT and X1.4H-450) were probed with trimethyl phosphorus (TMP), and ^31^P MAS SSNMR spectra were collected. Figure 4a shows a strong peak at −59 ppm in all of the samples, which can be assigned to physisorption of TMP.^53,54^ Interestingly, a detectable peak located from −4 to −5 ppm can be clearly seen in all Ru-loaded 13X zeolite samples (X1.4H, X1.4H-RT and X1.4H-450), which is attributed to the strong BAS (protonic in nature) to account for the chemical upshift.^55^ It is noticed that no such strong BAS peak can be identified by the probe SSNMR for the Na13X sample, suggesting that the Na^+^ ions have replaced nearly all of the H^+^ from the BAS. It is intriguing to see from Figure 4a that the BAS peak of X1.4H grows significantly in size after the NH_3_ decomposition at 450 °C (X1.4H-450). The results of ^1^H MAS SSNMR (Figure 4b) show three proton peaks for both X1.4H-RT and X1.4H-450, which correspond to 1.7 ppm (Si–OH), 2.4 ppm (Al–OH), and 3.4 ppm (BAS), respectively.^5,56^ It is clear from the figure that the proton signals on BAS (3.4 ppm) dramatically increase after the NH_3_ decomposition (X1.4H-450), which is indicative of their regeneration during the NH_3_ decomposition as compared to the X1.4H-RT sample. The increase of the proton peaks of BAS provided strongly endorsing support to the heterolytic cleavage of NH_3_ and the reformation of BAS via an FLP-type mechanism. It is noticed from the H_2_ temperature-programmed reduction (H_2_-TPR) result of the X1.4 sample (Figure S16) that consumption of H_2_ in the reduction process starts at about 340 °C. This suggests that the residue BAS H^+^ signals detected in X1.4H-RT by NPD and NMR in the prereduction process are still small, compared to the more facile proton regeneration (i.e., BAS) during NH_3_ decomposition, which is indicative of the favorable heterolytic cleavage via the FLP-type mechanism.

Further, in situ diffuse reflectance infrared Fourier transform spectroscopy (DRIFTS) experiments were conducted, where the X1.4H sample was allowed to preadsorb NH_3_ in an enclosed chamber at room temperature and then heated to 400 °C. Noticeably, strong detectable peaks at 1305 cm^–1^ (−OH formed on BAS O)^57^ and 3017 cm^–1^ (NH_4_^+^ ions formed by the chemisorption of NH_3_ on the BAS)^58^ are found to increase. At the same time, the peak at 3333 cm^–1^ (symmetrical υ of N–H in NH_3_)^10,58,59^ decreases (Figure 4d–f and Table S1), and the peak at 1625 cm^–1^ (coordinatively bonded NH_3_ species on Lewis acid sites)^60^ (Figures S4 and S5) disappears during the DRIFTS signal collections from time 0 to 120 min. This clearly shows the heterolytic cleavage of the N–H bond of NH_3_, which leads to the generation of NH_2_^–^ (on Ru) and BAS (H^+^ is regenerated on O), as the proposed FLP-type mechanism. Unreacted NH_3_ (free or weakly adsorbed) reacts with the regenerated H^+^ (of the BAS), therefore forming NH_4_^+^. Note that the peaks at 931 and 965 cm^–1^ are associated with gaseous-phase NH_3_ and/or physically adsorbed NH_3_.^59^ The reduction of these peaks indicates the consumption of NH_3_ during decomposition. The peak at 1207 cm^–1^ is associated with N–H stretching vibration modes of NH_3_ on Lewis acid sites, while the peaks at 1483 and 3280 cm^–1^ are associated with N–H stretching vibration modes of NH_4_^+^ species.^59,61^ Similarly, the in situ DRIFTS for the X1.4H sample was also performed under ND_3_ with the same experimental conditions (Figures S8 and S9), and the consumption of d-ammonia is also made with the confirmation of the corresponding H^+^/D^+^ regeneration on BAS.

NH_3_ molecules can adsorb on multiple surface sites with different extents depending on the acidity (NH_3_ can bind on acid sites strongly) of the sites. As a result, temperature-programmed desorption (TPD) of preadsorbed NH_3_ (see the NH_3_-TPD method in SI) on the pristine Na13X and Ru-loaded 13X zeolite sample after the NH_3_ decomposition (X1.4H-450) is assessed. As shown in Figure 4c, there is a broad and strong peak of NH_3_ desorption at 217 °C as well as two minuscule desorption peaks located at 435 and 500 °C. The low-temperature broad peak is assigned to the desorption of the weakly bound NH_3_, and the two small higher-temperature desorption peaks are assigned to the residue BAS, presumably corresponding to H2 and H1 sites in the pristine Na13X (TPD appears to be more sensitive than SSNMR in the detection of BAS). It is interesting to note that there is a slight temperature downshift and peak attenuation (low-temperature peak) of the partial Ru-exchanged X1.4H sample compared to the pristine Na13X, which may indicate that Ru can block up sites for NH_3_ uptake after the ion exchange and heat treatments (NH_3_-TPD in Figure 4c). However, it is apparent that the residue of two strong BAS peaks with originally low intensities (desorption temperatures of 435 and 500 °C) in the Ru-loaded 13X zeolite is increased in its magnitude after the NH_3_ decomposition. This observation is again consistent with all of the other characterizations, which also points to a heterolytic cleavage of NH_3_ over a Ru-loaded zeolite to regenerate strong BAS. The transmission electron microscopy (TEM) images show that the Ru-loaded 13X zeolites are large cubic-like shaped crystallites (Figures S20 and S21). The surfaces of all of the samples (X1.4, X1.4H and X1.4H-450) are apparently clean without any observed Ru NPs. This demonstrates that no RuO_2_ NPs formed on X1.4 after the Ru ion exchange, together with the differences of Ru EXAFS spectra of X1.4 and bulk RuO_2_ reference (Figure S12). This also indicates that no Ru NPs are aggregated during the H_2_ treatment or NH_3_ decomposition. High-angle annular dark-field scanning transmission electron microscopy (HAADF-STEM) was also performed, but unfortunately, we were unable to do the imaging for the 13X samples. These samples appeared to be extremely beam-sensitive: when the beam was focused upon the structure, we recorded the rapid collapse of such a porous structure. We attribute this to the fact that in the 13X zeolite after partial regeneration of the proton sites after hydrogen reduction and postreaction, the extensive BAS could make the sample highly unstable. In addition, the large 13X crystallites also render the imaging difficult to achieve. From the Brunauer–Emmett–Teller (BET) surface area analysis (Table S5), there is only a marginal change in the surface area and microporosity of X1.4 with or without heat treatment and postreaction. It is thus concluded that the porous structure is mostly preserved disregarding the treatments.

### DFT Calculations and Mechanisms

**Figure 5:**
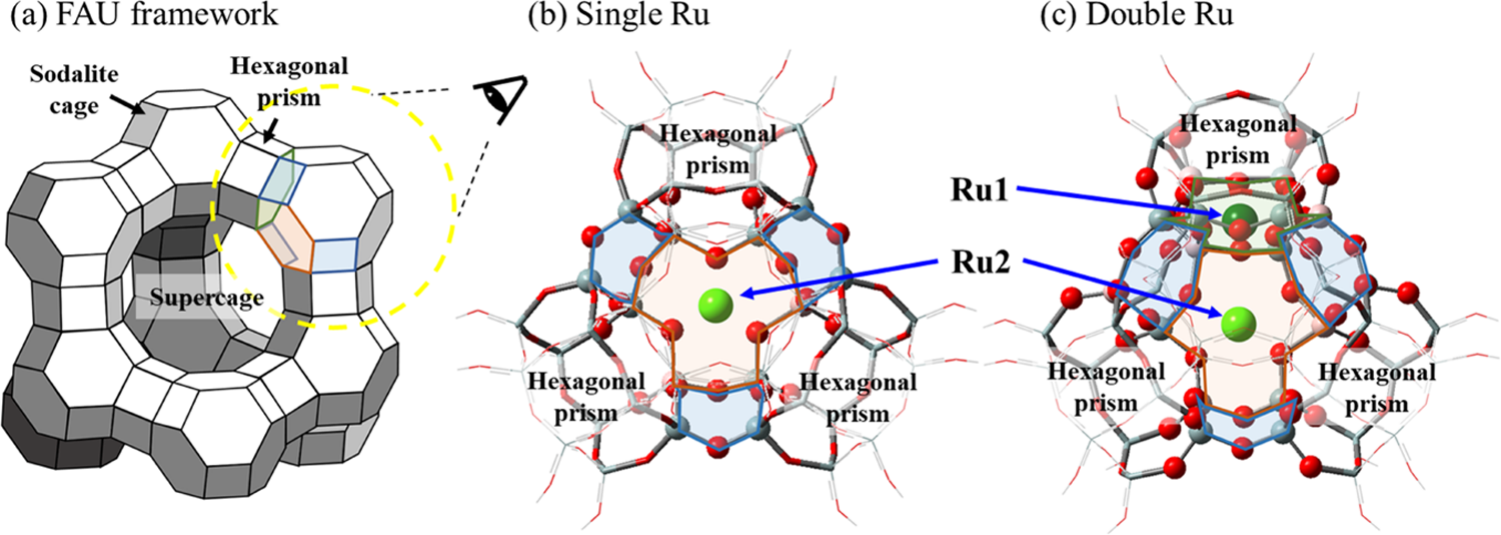
(a) Faujasite (FAU) framework structure. The four-membered rings shared between a solidate cage and hexagonal prisms are highlighted in blue. The six-membered ring shared between a sodalite cage and a supercage is highlighted in red, whereas the six-membered ring shared between a sodalite cage and the hexagonal prism is shown in green. Computational models of (b) single-Ru and (c) double-Ru catalysts as seen from the eye angle shown in panel (a). The Ru atom located at the center of the hexagonal ring is highlighted in red (Ru2). An additional Ru atom is located at the center of the hexagonal ring in the double-Ru catalyst, which is highlighted in green (Ru1). Balls, tubes, and wires represent atoms in the high, middle, and low ONIOM layers, respectively. The atoms are color coded according to the element (dark green = Ru1, light green = Ru2, red = O, gray = Si, pink = Al, and white = H).

It is thus clear from experiments that we observed a heterolytic cleavage of NH_3_ during the NH_3_ decomposition over a Ru-loaded zeolite, which is in sharp contrast to generally believed homolytic cleavage over metal NP.^8,62^ In order to appreciate the FLP-type N–H bond cleavage, computational chemistry calculations were carried out using a three-layer ONIOM scheme, as implemented in the Gaussian 09 software package^63^ (see the Computational Method in SI). The catalytic system was first constructed by introducing one or two Ru atom(s) to an aluminosilicate zeolitic cluster. The zeolite system was prepared from a 13X faujasite (FAU) crystalline structure obtained from the International Zeolite Association (IZA),^64^ and comprised one sodalite cage and four neighboring hexagonal prisms (Figure 5a). The marginal O atoms were saturated with protons, maintaining an overall neutral charge. In the single-Ru catalytic system (Figure 5b), one Ru is located at the center of a hexagonal ring that is shared between a sodalite cage and a supercage (i.e., Ru2). In the double-Ru catalyst (Figure 5c), another Ru was introduced to a neighboring hexagonal ring (i.e., Ru1). The sites that Ru metals occupy are identical to the ones characterized by the SXRD-Rietveld refinement experiments. Three and six framework Al atoms were included in the high ONIOM layers of the single- and double-Ru catalysts, respectively. Since one negative charge stems from each tetrahedrally coordinated Al, the formal oxidation number of all Ru atoms is found to be 3+, which is a common oxidation state of single-atom Ru complexes.^65−68^ The detailed ONIOM partitioning scheme is found in the SI. During geometry optimization, the high and mid layers were fully relaxed, while the atoms in the low layer were kept frozen. All geometries were verified as local minima or saddle points with the mode and number of imaginary vibrational frequencies, where the ground-state and transition-state structures have zero and one imaginary frequency, respectively. The optimized model structure is in good agreement with the experimentally derived core crystal structure according to our refinements from the corresponding SXRD/NPD data (Figures S33–S37 and Table S12). Enthalpy and entropy corrections were applied to the electronic energies to calculate Gibbs free energies at the experimental temperatures, as implemented in Gaussian 09. To assess thermodynamic stability of single-Ru catalysts at different oxidation states, the Gibbs free energies of H addition on the zeolite framework were also calculated. Specifically, up to three H atoms were added to regenerate BAS (on framework O atoms that were first neighbors to Al) in the optimized single-Ru catalyst, and the structures were reoptimized. The overall system charge remained neutral, thus modifying the Ru oxidation state. To calculate the reaction energy of BAS formation with modification of the Ru oxidation state, molecular hydrogen (H_2_) was used as a reference.

**Figure 6:**
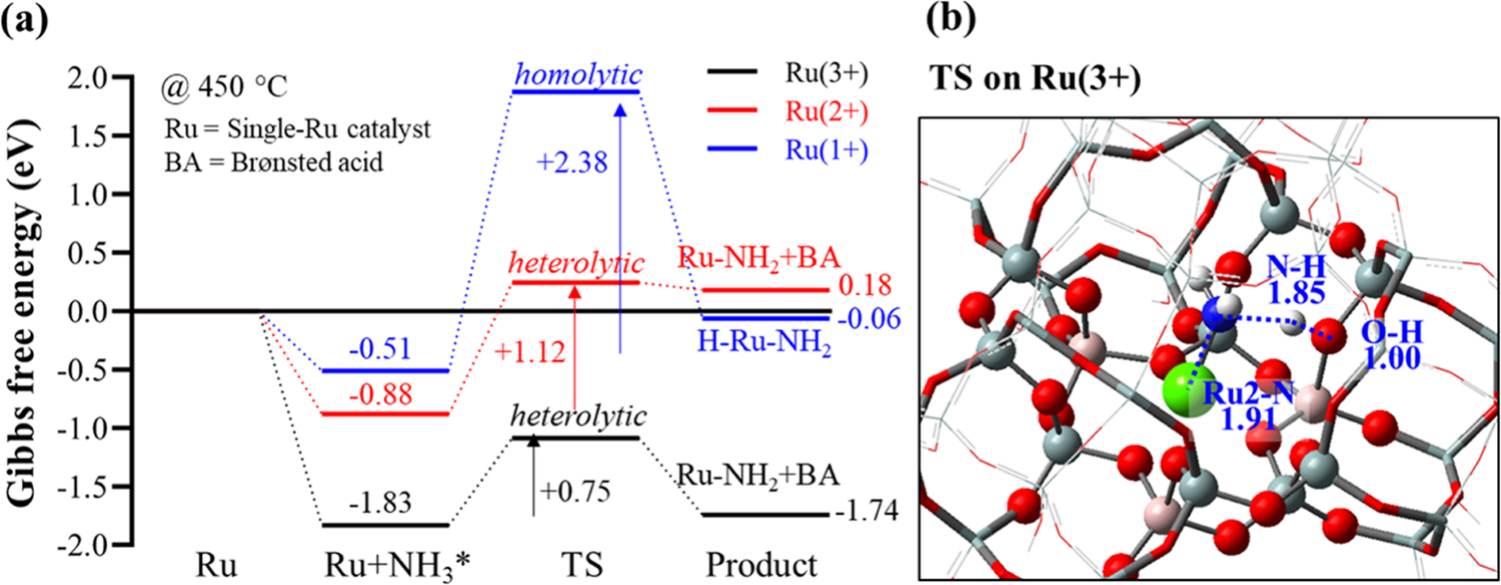
(a) Gibbs free energy profile for N–H dissociation on single-Ru catalysts at different oxidation states of Ru (3+, 2+, and 1+, associated with 0, 1, and 2 Brønsted acid site(s), respectively) calculated at 450 °C. An asterisk (*) denotes adsorbed species. (b) Optimized transition-state structure for heterolytic dissociation on Ru(3+). Selected interatomic distances are shown (in Å). The transition states on Ru(2+) and Ru(1+) are presented in the SI (Figure S27).

We first carried out theoretical calculations to understand the NH_3_ activation by a single Ru atom sitting in the zeolite cage. Figure 6 shows the Gibbs free energy profiles of NH_3_ activation on the single-Ru catalyst with three different oxidation states of Ru (1+, 2+, and 3+) calculated at 450 °C (experimental reaction temperature). The first step, NH_3_ adsorption, highly depends on the oxidation state of Ru. The binding energy is significantly stronger on Ru(3+) (−1.83 eV), which is comparable to that on a Cu-modified organic linker in metal–organic frameworks (MOFs) (−1.67 eV).^69^ Ru(2+) shows a moderate adsorption energy (−0.88 eV) that falls within a wide range of the reported values: −1.18 eV on the Mo_2_N(111) surface,^70^ −1.30 eV on HKUST-1 MOF,^71^ −0.84 eV on functionalized graphene,^72^ −0.72 eV on the Pt(111) slab,^73^ and −0.68 to −0.83 eV on Fe(110), Co(111), and Ni(111) metal surfaces.^74^ NH_3_ is relatively weakly bound on the low oxidation state Ru(1+) (−0.51 eV). Considering that NH_3_ is a probe molecule that interacts with acid sites, the adsorption energy results indicate that the acidity of Ru increases with its oxidation state. The next step is dissociation of the N–H bond of NH_3_. Both the homolytic and heterolytic N–H dissociations are explored on Ru(3+). The homolytic N–H dissociation results in the formation of a H–Ru–NH_2_ complex. On the other hand, the heterolytic dissociation leads to the formation of a Ru–NH_2_ complex and regenerated BAS in the zeolite framework. This is induced by a nearby but non-neighbor framework O that donates an electron pair to H^+^ of NH_3_ (i.e., Lewis base) and Ru that accepts an electron pair from NH_2_^–^ (i.e., Lewis acid), whereupon Ru is reduced. The O site belongs to the aluminate tetrahedra (AlO_4_^–^), which is electron rich. This activation resembles the functionality of FLPs^75,76^ as discussed, which has also been recently demonstrated in dihydrogen activation and alkane dehydrogenation in zeolite catalysis.^13,18,77^ It is noted that the energy required to carry out a metal–ligand cooperative activation mechanism^78^ for an O atom directly next to the Ru2 site (O2) is found to be too high, which is not favorable for the heterolytic cleavage of NH_3_. In contrast, for the O atom of the non-neighboring site (O3) to Ru2 (nonligand but spatially FLP-like), as represented in Figure 3d, the energy for cleavage is substantially lower to undergo the heterolytic cleavage for NH_3_. A similar case is also observed at Ru1 sites, where the energy of an O atom next to the Ru1 site (O3) is too high for the cooperative heterolytic cleavage, whereas the O atom of the non-neighboring site (O2) provides the site for the synergistic heterolytic cleavage for NH_3_. The heterolytic N–H dissociation on Ru(3+) is indeed found to be significantly more facile than the homolytic cleavage due to the lower activation energy (0.75 vs 3.48 eV) and reaction energy (0.09 vs 2.02 eV) involved (Figure S26). Likewise, Ru(2+) can heterolytically dissociate the N–H bond (Figure 6), with an energy barrier higher than that of Ru(3+) (1.12 eV), due to its weaker Lewis acidity. In contrast, the transition state and product structures corresponding to the heterolytic N–H dissociation on Ru(1+) could not be located. Instead, NH_3_ can undergo homolytic activation on Ru(1+) but with prohibitively a high activation energy (2.38 eV) (Figure 6). The initial N–H activation energies on Ru(3+) and Ru(2+) are comparable to the reported values in other heterogeneous catalyst systems in the literature: 1.82 eV on the Pt-covered TiO_2_(110) surface,^79^ 1.48 eV on the Fe nanocluster,^80^ 0.96 eV on Pt(111),^73^ 0.64 eV on the Mo_2_N(111) surface,^70^ and 0.72–1.11 eV on the Fe, Co, or Ni metal surface.^74^ Temperature effects on the heterolytic N–H dissociation on the single-Ru catalysts at the two oxidation states of Ru (3+ and 2+) suggest that the binding of NH_3_ is weakened, and the activation energy of N–H dissociation slightly increases with H^+^ to regenerate BAS, maintaining the overall energetic trends between the different oxidation states (Figure S28). The average activation energy (*E*_a_) determined experimentally based on the kinetic rate analyses of X1.4H at 30 000 mL g_cat_^–1^ h^–1^ is +94.4 kJ mol^–1^ (i.e., +0.98 eV) (Figure 1e), which matches very well with the computational results, with being closer to the barrier over Ru(2+).

**Figure 7:**
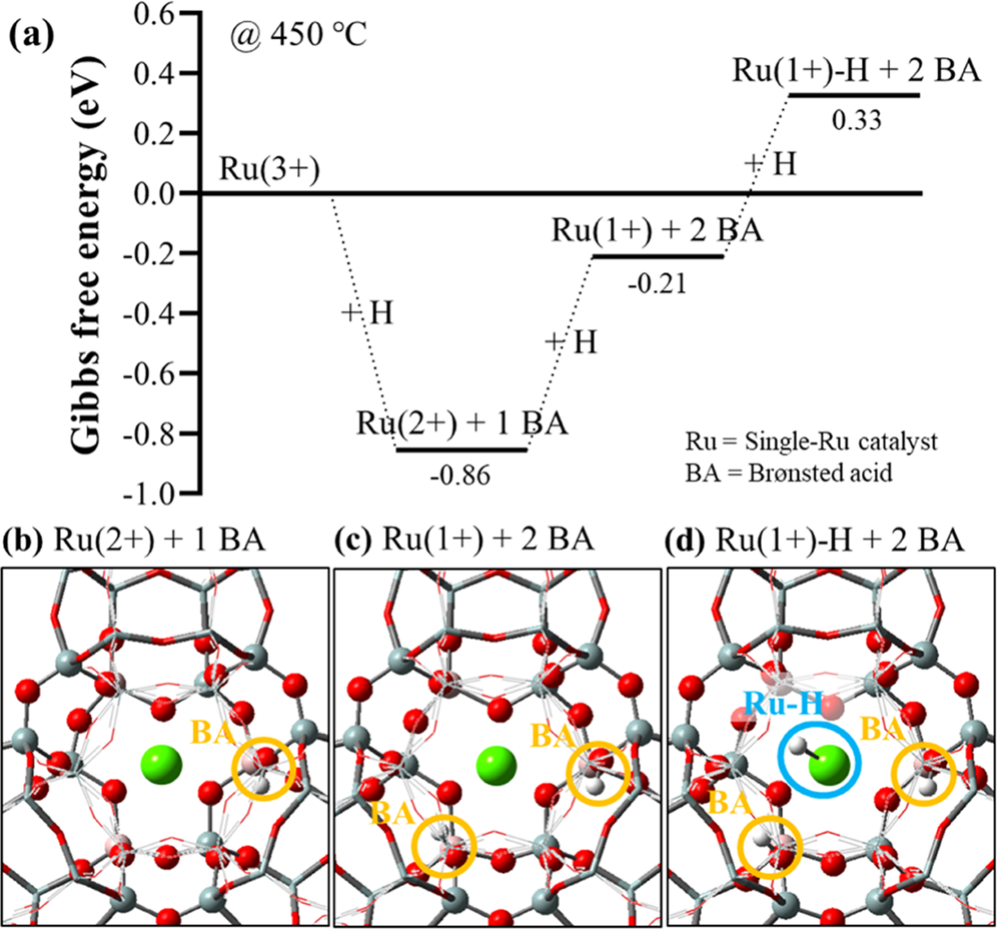
(a) Gibbs free energy profile for hydrogen addition to the single-Ru catalyst, calculated at 450 °C. The hydrogen molecule (H_2_) is used as a reference, and the addition of up to three hydrogen atoms is investigated gradually altering the oxidation state of Ru. (b–d) Optimized structure of each catalyst, where the Brønsted acid sites and the Ru–H complex are marked by circles (orange and blue, respectively).

It becomes clear that the oxidation state of Ru significantly affects the adsorption and activation of NH_3_, as well as the mode of activation. It is worth noting that the heterolytic N–H dissociation involves the reduction of the Ru center since a proton migrates from NH_3_ to the zeolite framework (i.e., BAS), compensating a negative charge from one tetrahedrally coordinated Al. Therefore, the effects of the oxidation state of Ru on the catalytic activity (Figure 6) can be partly attributed to the thermodynamic stability of the zeolite-stabilized Ru atom at each oxidation state. This is depicted in the hydrogen addition energetics in Figure 7, simulating a phase diagram of the active center at different oxidation states. The energetics of the sequential addition of the two hydrogen atoms (starting from Ru(3+)) shows that the most stable oxidation state of Ru in the zeolitic cluster is 2+, followed by 1+. This explains why Ru(3+) is more active than Ru(2+): upon heterolytic N–H dissociation, Ru(3+) is reduced to the more stable Ru(2+), whereas Ru(2+) is reduced to the less stable Ru(1+). On the other hand, the third hydrogen is initially introduced to the BAS (so that Ru becomes metallic), but moves to the Ru center during optimization, resulting in a Ru–H complex (Figure 7d). This indicates that the further reduction of Ru(1+) to Ru(0) is unfavorable, which explains why Ru(1+) cannot activate NH_3_ heterolytically, as shown in Figure 6a.

**Figure 8:**
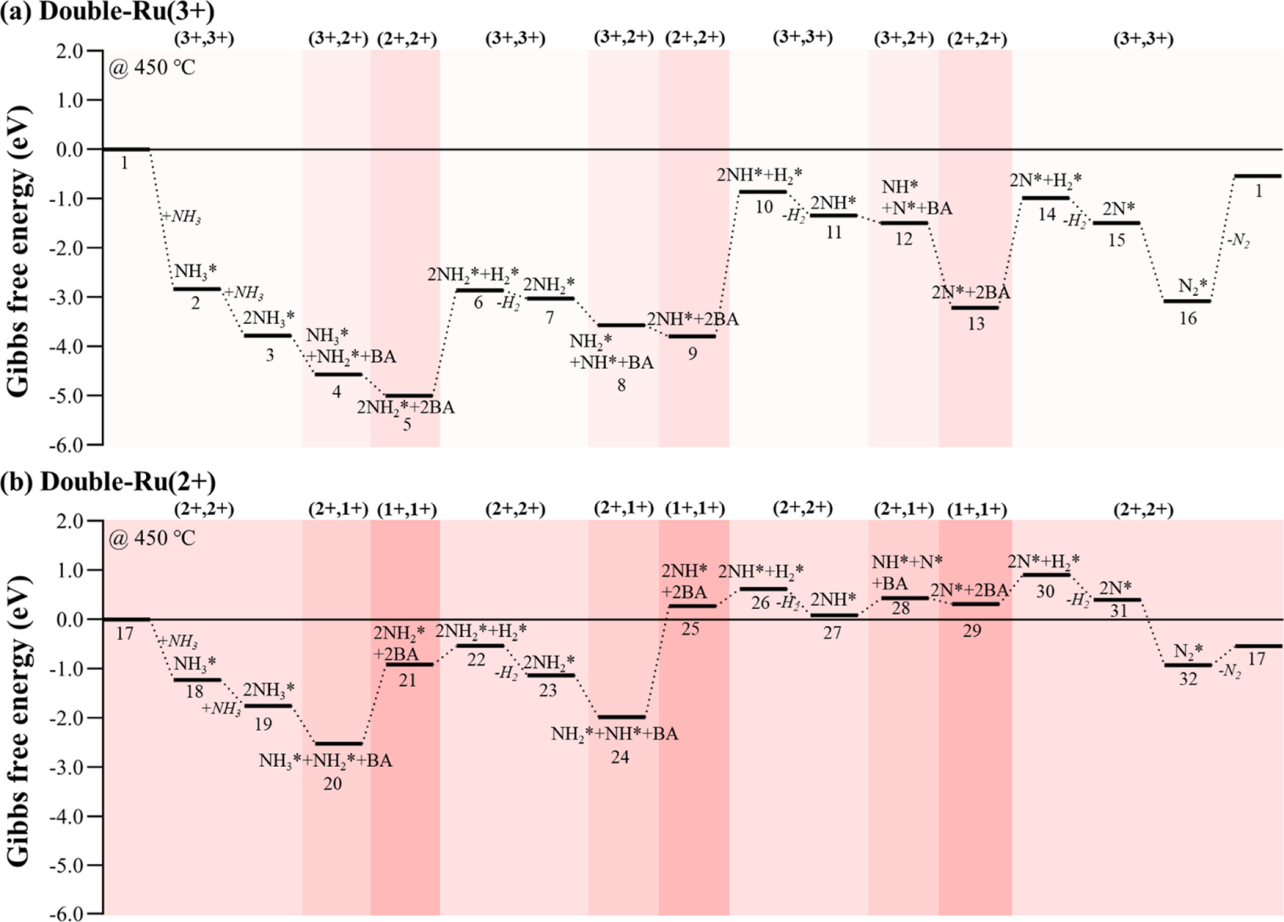
Gibbs free energy profile of full dissociation of two NH_3_ molecules (2NH_3_ → N_2_ + 3H_2_) on (a) double-Ru(3+) and (b) double-Ru(2+) catalysts, calculated at 450 °C. The formal oxidation states of the Ru atoms along the reaction coordinate are shown in parentheses. An asterisk (*) denotes adsorbed species on the catalysts. The optimized structures of each state are shown in the SI (Figures S29 and S30).

Next, we studied the reaction thermodynamics of a complete NH_3_ decomposition pathway (2NH_3_ → N_2_ + 3H_2_) on the double-Ru catalysts since the full NH_3_ decomposition reaction is likely to proceed over two Ru sites by means of dehydrogenation followed by recombination of N (to N_2_) and H (to H_2_).^70,74,81,82^ The reaction is investigated on the two different catalytic systems (Figure 8): double-Ru(3+) and double-Ru(2+) catalysts, since Ru(3+) shows more facile N–H activation, whereas Ru(2+) is in the most stable oxidation state under reaction conditions, according to Figure 7. Note that the double-Ru(2+) catalyst originates from the double-Ru(3+) catalyst by adding two hydrogens to generate two BAS. As expected, the double-Ru(2+) catalyst is found to be thermodynamically more stable than the double-Ru(3+) (by Δ*G* = 3.60 eV at 450 °C). On both catalysts, the reaction starts with the adsorption of two NH_3_ molecules, followed by the heterolytic N–H activation of each molecule. The NH_3_ molecules prefer being bound on each of the Ru centers than being coadsorbed on one Ru (states 3 and 19 in Figures S29 and S30, respectively). This suggests that recombination of N_2_ is indeed catalyzed by two single metal sites in close proximity, similar to the findings in homogeneous counterparts.^83^ On the double-Ru(3+) catalyst (Figure 8a), the adsorption and activation of NH_3_ show monotonically downhill energetics (1 → 5). The exothermicity of the heterolytic N–H dissociation steps (3 → 4, −0.79 eV and 4 → 5, −0.43 eV) can be attributed to the reduction of Ru to the most stable oxidation state (i.e., from 3+ to 2+). However, the reductive removal of two H^+^ to H_2_ is highly endothermic (5 → 6, 2.14 eV) as the Ru atoms are oxidized back to the relatively unstable high oxidation state (3+). Desorption of the generated H_2_ is mildly exothermic (6 → 7, −0.16 eV). Then, the same reaction steps (i.e., heterolytic N–H activation, H_2_ recombination and desorption) are repeated twice (7 → 11 and 11 → 15), exhibiting the same change in the oxidation states of Ru, and therefore, similar energetic trends. The catalytic cycle ends with the associative desorption of N atoms through an endothermic step (15 → 1, 0.95 eV). Considering the large energy span shown in the reaction thermodynamics (Figure 8a, Δ*G* = 4.46 eV between state 5 and the last state), the double-Ru(3+) is unlikely to be responsible for the high activity of the catalyst. On the other hand, the double-Ru(2+) catalyst shows more milder reaction thermodynamics (Figure 8b). The NH_3_ adsorption and first N–H dissociation steps are favorable (17 → 20, −2.52 eV), whereas the second NH_3_ activation step is endothermic (20 → 21, 1.61 eV) as the Ru atoms are in the less stable Ru(1+) state. The reductive removal of two H^+^ is mildly exothermic (21 → 23, −0.22 eV), which is in contrast to the highly endothermic equivalent step in the +3 state of the Ru (5 → 7). To complete the full decomposition reaction, the same steps (i.e., N–H activation and associative H_2_ desorption) are repeated twice, followed by associative desorption of N_2_, as also shown in Figure 8a. In this Ru(2+) catalytic cycle, the largest thermodynamic energy span is 3.43 eV (between states 20 and 30). Further, we investigated a reaction that involves formation of a hydrazine (N_2_H_4_) intermediate on the double-Ru(2+) catalyst (Figures S31 and S32). The significantly large thermodynamic energy span (4.24 eV) indicates that N–N coupling is not likely to occur until the N–H bonds are fully dissociated. Overall, the reaction thermodynamics (Figure 8) clearly demonstrate the role of the Ru oxidation state in the NH_3_ decomposition reaction, attributing the activity of the catalyst to the thermodynamically stable Ru(2+) centers, rather than the unstable Ru(3+) centers. This dynamic change in the oxidation states of Ru is responsible for the generation and depletion of BAS on the zeolite framework, giving rise to the FLP catalyst functionality. The most probable oxidation state of the Ru centers (2+) is confirmed by the XANES analysis of the rapidly quenched catalyst (X1.4H-450) from NH_3_ decomposition (Figure S10). The results clearly indicate that the average oxidation state of the working catalyst of lower energy shifts from the Ru(3+) edge toward the reference of Ru(0), which is agreeable to the edge position of Ru(2+).^84^

## Conclusions

A Ru-loaded 13X zeolite was synthesized, tested for NH_3_ decomposition, and characterized in detail. It is revealed for the first time that this Ru-loaded zeolite provides a more superior specific activity in NH_3_ decomposition than most Ru-containing catalysts reported in the literature. The isolated Ru sites confined in the 13X zeolite are elucidated by SXRD and XAS. It is evident that such isolated Ru species combined with the nearby H^+^-depleted O sites from BAS can activate NH_3_ synergistically by an FLP-type mechanism and regenerate the proton sites, as shown by the NPD-Rietveld refinement, SSNMR, in situ DRIFTS, NH_3_-TPD, and computational chemistry calculations. As shown in Table 1 in the Results and Discussion section, there have been a lot of studies being placed on Ru nanoparticles on different supports, and the typical mechanism over the extended metal surface of a nanoparticle is carried out by the homolytic cleavage of NH_3_ to N_2_ and H_2_ over the extended metal sites. Thus, searching for a new class of catalysts with different reaction mechanism(s) may give us a novel way to carry out the reaction. This mechanism over isolated Ru in the zeolite as FLP synergetic sites clearly differs from the classical coordinated stepped Ru sites in cluster/NP and gives insights for designing better catalyst candidates with higher catalytic atom efficiencies.^62,85^ This class of novel catalysts apparently achieves superior catalytic performance in NH_3_ decomposition with a lower apparent kinetic barrier under comparable reaction conditions. Importantly, our experimental and theoretical studies demonstrate a synergistic effect of Ru^2+^ pairs in confined space in the zeolite, resulting in cycles of N–H activation and BAS depletion/regeneration. Our finding suggests the importance of the utilization of synergistic single-atom noble catalysts in NH_3_ decomposition in zeolites, which can efficiently use the noble metals to the greatest extent. Thus, stabilizing FLP in zeolites can provide new opportunities for designing synergistic catalysts for other related catalysis.

## Supporting Information Available

Experimental details, methods, computational method, and structure refinement data including Figures S1–37 and Tables S1–12 (PDF)
