# Supplementary material for: Confined Ru Sites in a 13X Zeolite for Ultrahigh H2 Production from NH3 Decomposition
Source: J Am Chem Soc. 2023 Jun 21;145(26):14548–61. doi: 10.1021/jacs.3c05092 (PMC10326886; doi:10.1021/jacs.3c05092)
Supplement: Supplementary file 1 — ja3c05092_si_001.pdf [file ja3c05092_si_001.pdf]

Supporting Information

Confined Ru sites in 13X zeolite for ultra-high H_2 production from NH_3 decomposition

Kwan Chee Leung[1], Sungil Hong[2], Guangchao Li[1,3], Youdong Xing[3], Bryan Kit Yue Ng[1], Ping-Luen Ho[1], Dongpei Ye[1], Pu Zhao[1], Ephraem Tan[1], Olga Safonova[4], Tai-Sing Wu[5], Molly Meng-Jung Li[3], Giannis Mpourmpakis [2*] and Shik Chi Edman Tsang[1*]

[1]Wolfson Catalysis Centre, Department of Chemistry, University of Oxford, OX1 3QR, UK
[2]Department of Chemical Engineering, University of Pittsburgh, Pittsburgh, PA 15261, USA
[3]Department of Applied Physics, Hong Kong Polytechnic University, Hong Kong
[4]Paul Scherrer Institut WLGA/217, Forschungsstrasse 111, 5232 Villigen PSI, Switzerland
[5]National Synchrotron Radiation Research Center, Hsinchu 30076, Taiwan

*Correspondences' emails: edman.tsang@chem.ox.ac.uk (Shik Chi Edman Tsang); gmpourmp@pitt.edu (Giannis Mpourmpakis)

Materials and Methods

Synthetic method and H_2 pre-treatment

Ruthenium was exchanged into the sodium-type 13X zeolite (Na13X, Alfa Aesar) via ion-exchange method. The unit cell formula of the 13X zeolite is $Na_{96}Al_{96}Si_{96}O_{384}$. The Ru ions is +3 oxidation, the theoretical maximum Ru loading would be 32 Ru^{3+} ions in one 13X unit cell hence, 22.05 wt.%, assuming all of the 96 Na^+ sites in one 13X unit cell are replaced by Ru^{3+} ions. Ruthenium chloride hydrate ($RuCl_3 \cdot xH_2O$, Fluorochem) was dissolved in DI water and Na13X zeolite was added into the solution. The mixture was stirred and heated at 80 °C for 2 hours. It was then washed by water with sonication several times. The sample was put into oven at 80 °C overnight to allow the sample to be dried. Further ruthenium chloride hydrate was added into the sample and repeated the above processes for stepwise ion-exchange, in order to increase the Ru-loading of the samples. The dried Ru-loaded 13X zeolites were activated at 110 °C at a ramp rate of 5 °C min^{-1} under 5% H_2/Ar for 2 hours, followed by 350 °C at a ramp rate of 1 °C min^{-1} for 6 hours.

Catalytic studies

The ammonia decomposition was performed in a specialized quartz-lined stainless-steel fix-bed reactor. Pelletizing and sieving were performed before the ammonia decomposition, with the use of sieve value of 100 mesh. The reactor was positioned at the centre of the furnace and attached to the gas inlet, with an extra thermocouple and back pressure readout to make sure that there was no deviation of the temperature of the sample from the reactor temperature with no creation of backpressure. 50.0 mg of catalyst was loaded into a quartz tube (4 mm i.d.) and put into the reactor. Temperature was then raised to the desired value at a ramp rate of 5 °C min^{-1} and evaluated under the identical flow conditions in ammonia gas (NH_3). The resultant produced gases were analysed through an *in-situ* way via a direct-connected gas chromatography (GC) which contains two thermal conductivity detectors (TCD). Ammonia was decomposed in the following conditions: i) 30,000 mL g_{cat}^{-1} h^{-1} at 400°C, ii) 15,000 mL g_{cat}^{-1} h^{-1} at 400°C, iii) 30,000 mL g_{cat}^{-1} h^{-1} at 450°C and iv) 15,000 mL g_{cat}^{-1} h^{-1} at 450°C.

Equations

1. **Specific Activity**

$$\text{WHSV} \times \frac{L}{1000mL} \times \frac{mole}{24L} \times \frac{conversion\,\%}{wt\%/molar\ mass\ of\ Ru}$$

$$= 30000 \times \frac{mL}{g \times h} \times \frac{L}{1000mL} \times \frac{mole}{24L} \times \frac{conversion\,\%}{wt\%} \times 101.07 \times \frac{g}{mole}$$

$$= \frac{30000 \times 101.07}{1000 \times 24} \times \frac{conversion\,\%}{wt\%} \times h^{-1}$$

Synchrotron X-ray diffraction (SXRD)

SXRD data of Ru-loaded 13X zeolite were collected at beamline X04SA at Paul Scherrer Institut, Switzerland. Samples were loaded into borosilicate capillaries of diameter 0.5 mm and scanned at room temperature. All structural refinements and electron-density visualisation were carried out in TOPAS and VESTA.

Neutron powder diffraction (NPD)

NPD of samples (1) X1.4H with ammonia adsorption at room temperature (X1.4H-RT) and (2) X1.4H with ammonia decomposition at 450 °C (X1.4H-450) were performed via High-Resolution Powder Diffractometer for Thermal Neutrons (HRPT) at SINQ neutron source from Paul Scherrer Institut, Switzerland.

Temperature-programmed Analysis

Temperature-programmed desorption (TPD) and Temperature-programmed reduction (TPR) experiments were carried out in automated flow chemisorption analyser (ChemBET Pulsar). The sample was loaded in a double-layer wall quartz reactor, where the two ends were connected to a Swagelok fitting to allow the reactant gases to pass through the catalyst bed. The inlet gas stream introduced at the inner layer reactor (top to bottom) passed through the catalyst bed and ejected from the outer side of the reactor (bottom to top) to release the gases. The product stream was sampled continuously using a thermal conductivity detector (TCD). The reactor temperature was controlled by a heating furnace, and the temperature readings were recorded simultaneously with the TCD signals.

For NH_3-TPD, the sample was pre-treated at 300 ◦C for 1 h under pure He gas to remove any impurity or water from the surface. The temperature was then decreased to 40 ◦C and 10 % NH_3/He was fed into the sample bed for 1 h to allow the adsorption of NH_3 on the sample. The gas was replaced with He and maintained until a stable TCD baseline was obtained. NH_3-TPD analysis was conducted while the reactor temperature increased to 700 ◦C at a ramp rate of 10 ◦C/min.

For H_2-TPR, the sample was first pre-treated at 300 ◦C for 1 h under pure He gas to remove any impurity or water from the surface. The temperature was then decreased to 40 ◦C and maintained until a stable TCD baseline was obtained. After switching the gas to 5% H_2/Ar, H_2-TPR analysis was conducted while the temperature was increased to 600 ◦C at a ramp rate of 5 ◦C/min.

For N_2-TPD, the sample was pre-treated at 350 ◦C for 1 h under 5% H_2/Ar gas for pre-treatment. The temperature was then decreased to 40 ◦C and pure N_2 was fed into the sample bed for 1 h to allow the adsorption of N_2 on the sample. The gas was replaced with He and maintained until a stable TCD baseline was obtained. N_2-TPD analysis was conducted while the reactor temperature increased to 700 ◦C at a ramp rate of 5 ◦C/min.

Solid state nuclear magnetic resonance (SSNMR)

All the NMR experiments were performed on a Jeol ECZ500R 500 MHz Solid-State NMR spectrometer operating at a Larmor frequency of 495.13 and 200.43 for the ^{1}H and ^{31}P nucleus, respectively. Solid state ^{1}H and ^{31}P NMR spectra were recorded using a 3.2 mm magic-angle-spinning (MAS) probe operating at a spinning rate of 10kHz and 12 kHz, respectively. A single-pulse sequence with a $\pi/2$ pulse length of 2.53 μs and a recycle delay of 5 s was used for the ^{1}H NMR experiments. 1D ^{31}P MAS NMR experiments were conducted with a $\pi/2$ pulse length of 2.62 μs and a high-power proton (^{1}H) decoupling using a $\pi/2$ pulse of 2.53 us and a recycle delay of 15 s. The chemical shift of the ^{1}H and ^{31}P nucleus was externally referenced to adamantane and $NH_4H_2PO_4$ (0.81 ppm).

For acidity characterization by means of the ^{31}P-trimethylphosphine (TMP) NMR approach, sample was placed in a glass tube and connected to a vacuum manifold for the dehydration treatment prior to the adsorption of TMP probe molecule. Typically, the dehydration treatment was carried by gradual heating at a ramp rate of 1 °C/min until reaching the final temperature (400 °C), then maintained at the temperature for at least 10 h under vacuum, then cooled to ambient temperature. Subsequently, a known amount of volatile TMP molecule was transfer onto the sample frozen over a liquid N_2 bath. Then, the sample was evacuated at room temperature for 1 h to remove physisorbed TMP molecules. Prior to the ^{31}P NMR experiments, the sample was transferred into a ZrO_2 rotor, then sealed with a Vespel MAS cap in a nitrogen glovebox.

Diffuse reflectance infrared fourier transform spectroscopy (DRIFTS)

In-situ DRIFTS experiments were performed on Nicolet iS50 FT-IR spectrometer with a high temperature, high pressure DRIFTS reaction cell using an MCT/A detector and at a resolution of 4 cm^{-1}. *In-situ* DRIFTS of ammonia decomposition experiments, 50 mg of sample was first activated in pure N_2 flow (10 mL/min) at 400 °C (with linear heating to 400 °C for 2 h and holding for 4 h). The system was then cooled down to room temperature (RT) and the sample was scanned using KBr background. After taking the background spectrum under N_2 atmosphere at RT, a constant 10% NH_3 flow (20 mL/min) were introduced for 20 min. The flow was then turned off and both inlet and outlet of the sample holder were closed. Scanning was performed at RT, 100 °C, 200 °C, 300 °C and 400 °C, using the background that the sample itself scanned under N_2 at RT. Extra scanning was done after the temperature reached 400 °C at 0 min, 15 min, 30 min, 45 min, 60 min and 120 min. Each spectrum was scanned 64 times.

Brunauer-Emmett-Teller (BET) surface area analysis

N_2 adsorption isotherm measured at -196 °C and up to 1 bar was recorded on a Micromeritics Tristar instrument. All sorption isotherms were obtained using ultrahigh purity gases (99.999%). Before the sorption analysis, a sample (approximately 70 mg) was loaded into a sample cell and subjected to a dynamic vacuum of 10−5 Torr at 200 °C for 16 hours. N_2 adsorption data with an initial slope (0.01 to 0.1 P/P_0) permitted the calculation of the apparent surface areas based on the Brunauer-Emmett-Teller (BET) equations. Pore size distribution

(PSD) was calculated by DFT embedded in the Micromeritics software. A model used in the PSD analysis is based on a cylindrical pore structure with an oxide surface analysed by N_2 at -196 °C.

X-ray absorption spectroscopy (XAS)

The XAS data were collected at Beamline BL07A the Taiwan Light Source at National Synchrotron Radiation Research Center, Taiwan. A Si(111) double crystal monochromator was used to scan the photon energy.

Transmission electron microscopy (TEM) and high-angle annular dark-field scanning transmission electron microscopy (HAADF-STEM)

High-resolution transmission electron microscopy images were taken by JEOL JEM-2100 and annular dark-field contrast by JEOL ARM-200F analytical electron microscopes operating at 200 kV for imaging and characterisation. The bright-field imaging was recorded using a Gatan Orius charge-coupled device (CCD) camera, the imaging can provide an amplitude contrast resulting from variations in mass or thickness. The contrast of ADF imaging directly corresponded with elements with a higher Z number, more electrons are scattered at higher angles due to greater electrostatic interactions between the nucleus and electron beam.

Computational Method

Computational chemistry calculations were performed using the ONIOM scheme[1-4] as implemented in Gaussian 09 software package[5]. The ONIOM method can accurately describe chemistry on zeolite frameworks with a reasonable computational cost[6-9]. Each structure is composed of three ONIOM layers that were treated with different computational methods: high-level theory layer with the ωB97XD functional[10], mid-level theory layer with semi-empirical PM6 method[11], and low-level theory layer with the universal force field method[12]. In the high-level theory layer along with the ωB97XD functional, the 6-31G(d) basis set was used for Si, Al, O, N, and H atoms, and the Los Alamos LANL2DZ effective core pseudo-potentials[13-15] was used for Ru, which has been shown to describe heavy atoms well by accounting for relativistic effects[16]. To build the single-Ru(3+) catalyst model (Figure S27), the Ru atom, framework atoms (i.e., Si, Al, and O) of a 6-membered ring close to Ru, as well as the first neighbours of the 6-membered ring are included in the high ONIOM layer. Additionally, 9 more framework atoms are included in the high layer since the catalyst was distorted during the preliminary optimization calculations without their inclusion. The mid layer includes the first two nearest neighbour atoms from the edge of the high layer plus the atoms that are linked to two mid-layer atoms. The rest of the atoms comprise the low layer, resulting in 34, 42, and 105 atoms in the high, mid, and low layers. In the double-Ru(3+) catalyst model, the high ONIOM layer includes two Ru atoms, framework atoms of two 6-membered rings, as well as the first neighbours of the rings (Figure S23). With further inclusion of the linking atoms and 4 additional tetrahedra (SiO_4) based on the preliminary calculations, 61 atoms are assigned to the high layer. The first neighbours and their linking atoms are included in the mid layer (39 atoms), and the remaining atoms are in the low layer (82 atoms).

The catalyst models in a lower oxidation states of Ru (2+) are constructed by adding Brønsted acid sites. Any adsorbed species and Brønsted acid sites are treated with the high-level theory.

Supplementary Results

SXRD

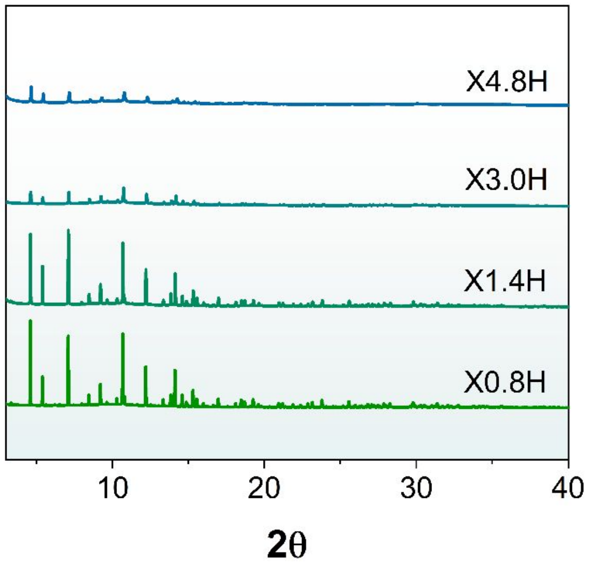

Figure S1. SXRD of the Ru-loaded 13X zeolites after H_2 pre-treatment, collected at X04SA at Paul Scherrer Institut, Switzerland.

XRD

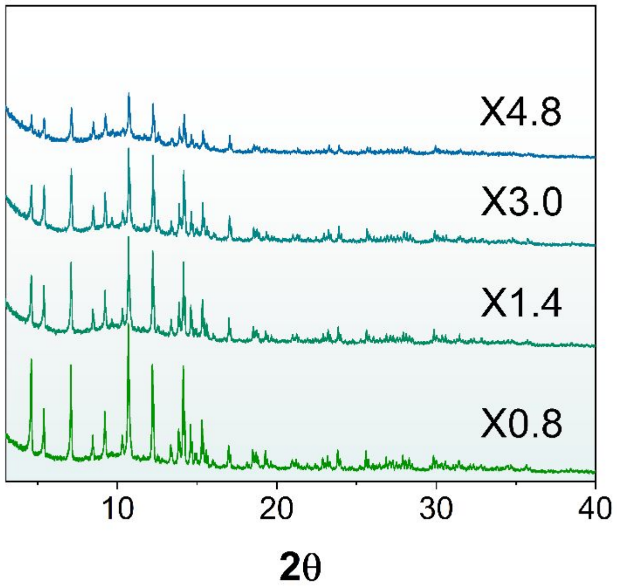

Figure S2. XRD of the Ru-loaded 13X zeolites before H_2 activation (X0.8, X1.4, X3.0 and X4.8). The XRD data were collected via monochromatic Mo anode source (λ = 0.7107 Å).

DRIFTS

DRIFTS spectra of X1.4H and pristine Na13X

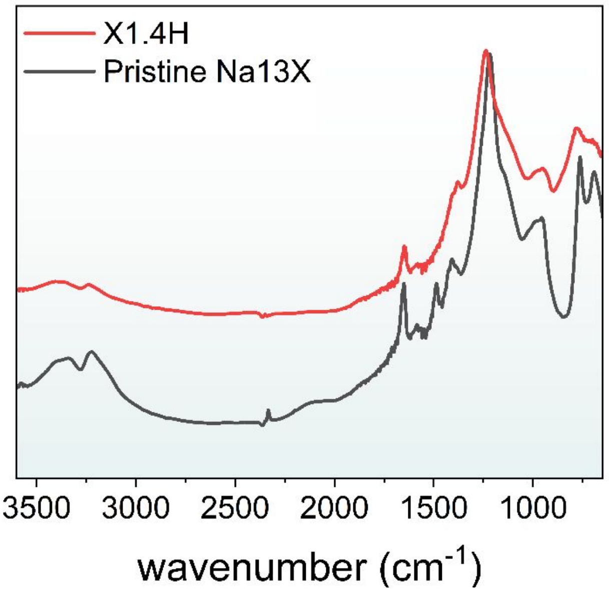

Figure S3. Full DRIFTS spectra of X1.4H and pristine Na13X in N_2 at RT, using KBr as background.

In-situ DRIFTS spectra of X1.4H with adsorbed NH_3

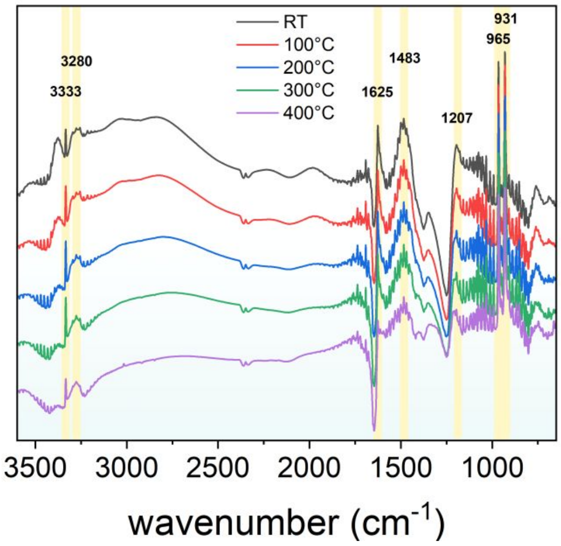

Figure S4. Full *in-situ* DRIFTS spectra of X1.4H from RT to 400 °C after NH_3 adsorption at RT, using X1.4H in N_2 at RT as background.

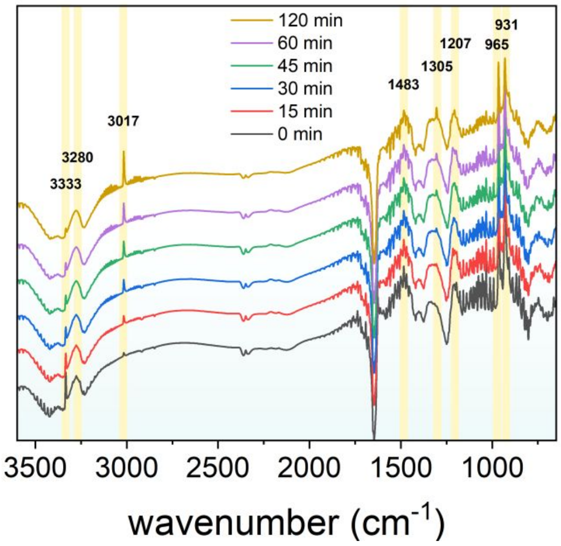

Figure S5. Full *in-situ* DRIFTS spectra of X1.4H from 0 min to 120 min at 400 °C after NH_3 adsorption at RT, using X1.4H in N_2 at RT as background.

***In-situ* DRIFTS spectra of Na13X with adsorbed NH_3**

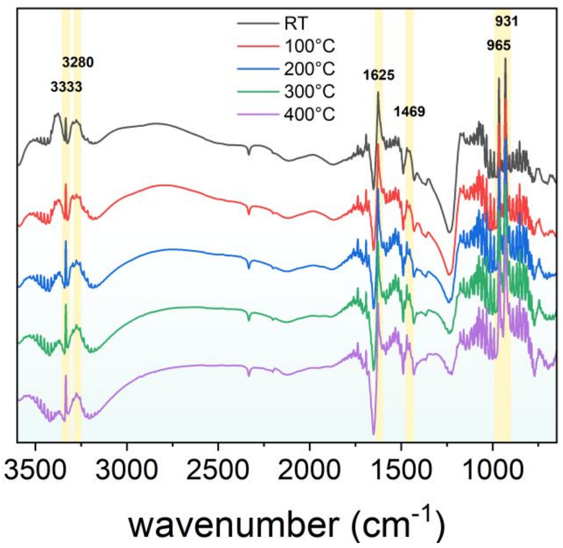

Figure S6. Full *in-situ* DRIFTS spectra of pristine Na13X from RT to 400 °C after NH_3 adsorption at RT, using pristine Na13X in N_2 at RT as background.

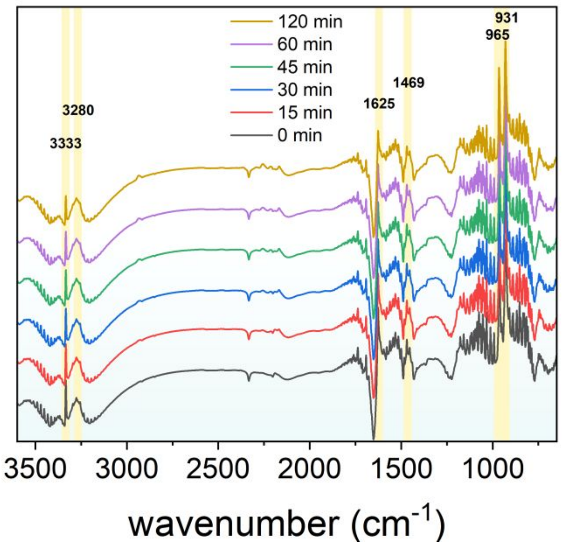

Figure S7. Full *in-situ* DRIFTS spectra of pristine Na13X from 0 min to 120 min at 400 °C after NH_3 adsorption at RT, using pristine Na13X in N_2 at RT as background.

Table S1. Position and assignment of the IR bands and reference

Wavenumber (cm^{-1})	Assignment	Reference
3333	Symmetrical υ of N-H in NH_3 on Lewis acid sites	[17-19]
3280	N-H stretching vibration modes of NH_4^+ species	[19, 20]
3017	NH_4^+ ions formed by the chemisorption of NH_3 on the BAS	[18]
1625	Coordinatively bonded NH_3 species on Lewis acid sites	[21]
1483	N-H stretching vibration modes of NH_4^+ species	[19, 20]
1305	-OH formed on BAS O	[22]
1207	N-H stretching vibration modes of NH_3 on Lewis acid sites	[19, 20]
965, 931	Gaseous-phase ammonia and/or physically adsorbed NH_3	[19]

In-situ DRIFTS spectra of X1.4H with adsorbed ND_3

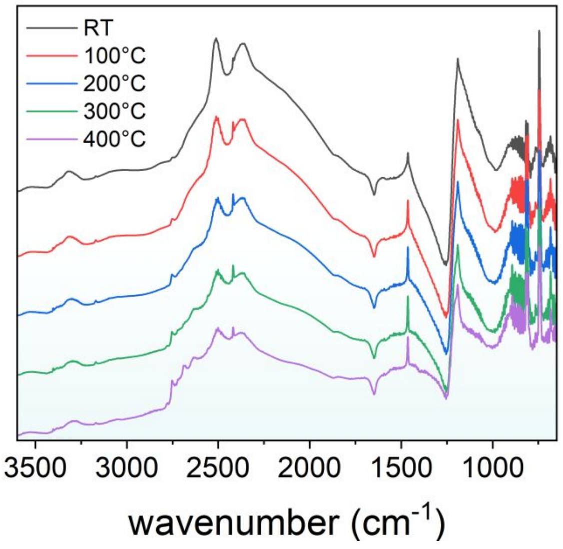

Figure S8. Full *in-situ* DRIFTS spectra of X1.4H from RT to 400 °C after ND_3 adsorption at RT, using X1.4H in N_2 at RT as background.

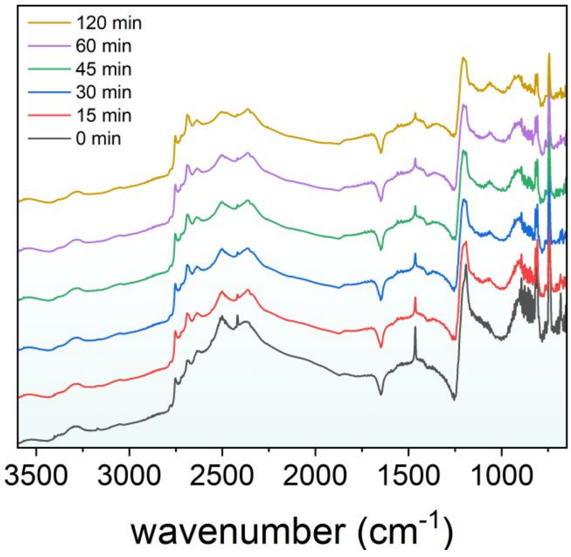

Figure S9. Full *in-situ* DRIFTS spectra of X1.4H from 0 min to 120 min at 400 °C after ND_3 adsorption at RT, using X1.4H in N_2 at RT as background.

XAS

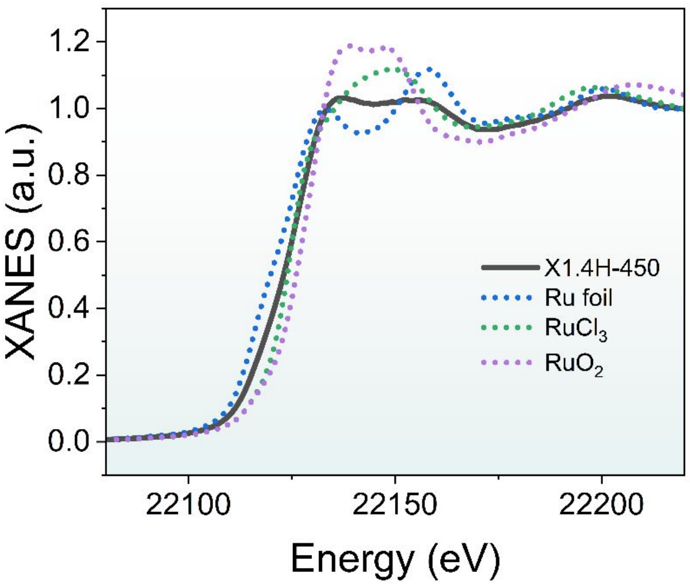

Figure S10. The XANES of quenched X1.4H-450 from NH_3. The oxidation state is between +3 and 0.

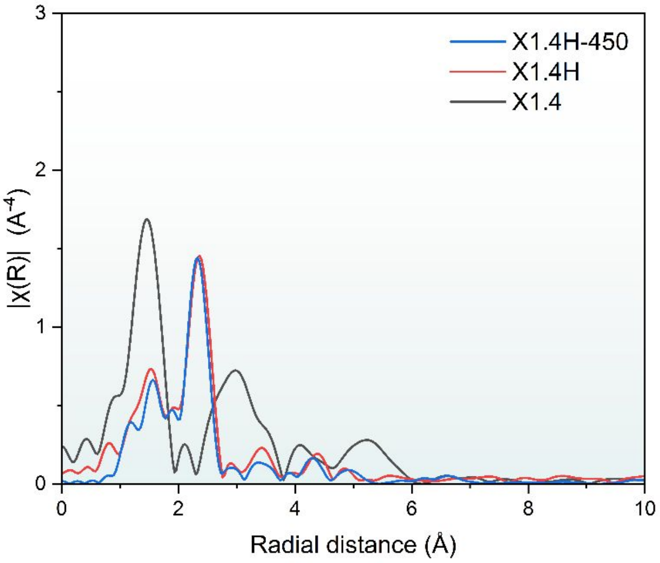

Figure S11. Fourier Transformed of Ru EXAFS spectra for X1.4, X1.4H and X1.4H-450.

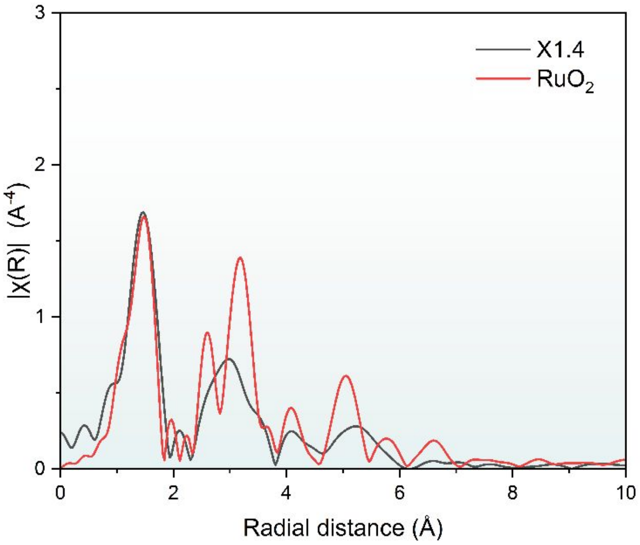

Figure S12. Fourier Transformed of Ru EXAFS spectra for X1.4 and bulk RuO_2 reference.

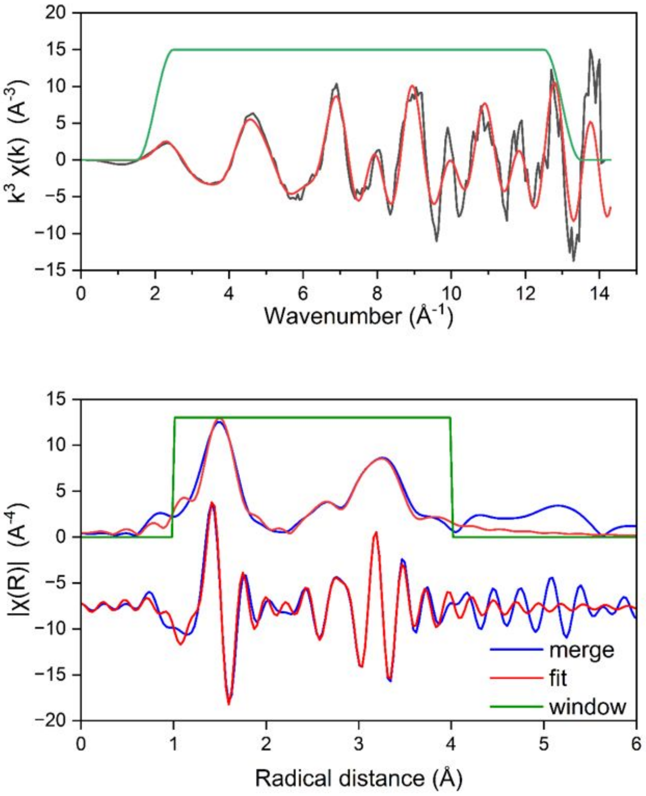

Figure S13. Ru EXAFS and curvefit in *k*-space (k^3 weighted) and *R*-space (k^3 weighted) of X1.4.

Table S2. The curvefit parameters of EXAFS of X1.4. S_0^2 was fixed at 0.733 as evaluated from a fitting of the EXAFS data of RuO_2. ΔE_0(O) was evaluated as -2.42 ± 0.94 eV. ΔE_0(Ru) was evaluated as -2.91 ± 4.80 eV. Data range $2.0 \leq k \leq 13$ Å^{-1}, $1.0 \leq R \leq 4.0$ Å. R factor for the fit is 1.73%. The fitting results is based on the $P4_2/m$ nm structure of RuO_2.

Path	CN	R/Å	σ^2 / 10^{-3}
Ru-O	5.91 ± 0.46	1.963 ± 0.007	2.0 ± 1.0
Ru-Ru	2.00 ± 0.01	3.12 ± 0.06	3.6 ± 1.6

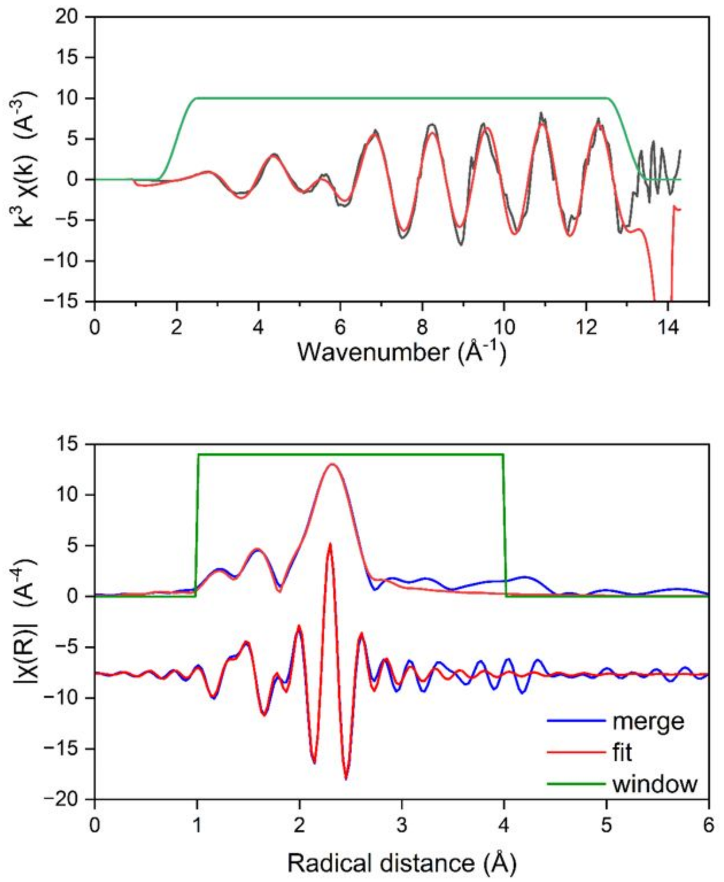

Figure S14. Ru EXAFS and curvefit in *k*-space (k^3 weighted) and *R*-space (k^3 weighted) of X1.4H.

Table S3. The curvefit parameters of EXAFS of X1.4H. S_0^2 was fixed at 0.733 as evaluated from a fitting of the EXAFS data of RuO_2. ΔE_0(O) was evaluated as -5.89 ± 4.76 eV. ΔE_0(Ru) was evaluated as -7.86 ± 1.18 eV. Data range $2.0 \leq k \leq 13$ Å^{-1}, $1.1 \leq R \leq 4.0$ Å. R factor for the fit is 2.98%. The fitting results is based on the $P4_2/m$ nm structure of RuO_2.

Path	CN	R/Å	σ^2 / 10^{-3}
Ru-O	3.04 ± 1.32	1.99 ± 0.03	7.1 ± 5.1
Ru-Ru	4.90 ± 0.77	2.668 ± 0.006	4.7 ± 0.9

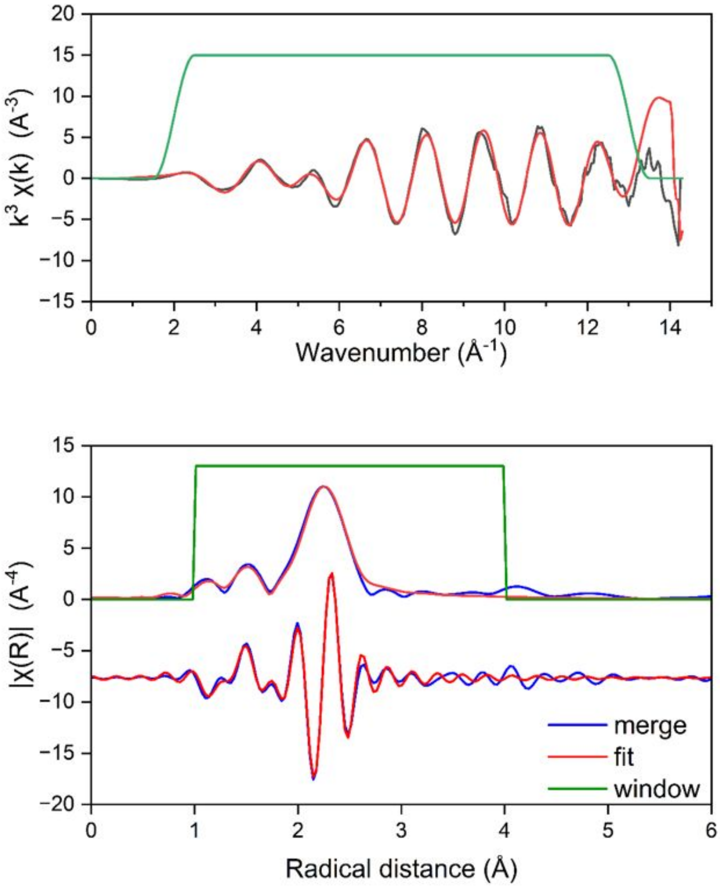

Figure S15. Ru EXAFS and curvefit in *k*-space (k^3 weighted) and *R*-space (k^3 weighted) of X1.4H-450.

Table S4. The curvefit parameters of EXAFS of X1.4H-450. S_0^2 was fixed at 0.733 as evaluated from a fitting of the EXAFS data of RuO_2. ΔE_0(O) was evaluated as 0.54 ± 6.10 eV. ΔE_0(Ru) was evaluated as -6.66 ± 1.21 eV. Data range 2.0 ≤ k ≤ 13 $Å^{-1}$, 1.0 ≤ R ≤ 4.0 Å. R factor for the fit is 2.40%. The fitting results is based on the $P4_2/m$ nm structure of RuO_2.

Path	CN	R/Å	σ^2 / 10^{-3}
Ru-O	2.43 ± 1.55	1.99 ± 0.05	9.3 ± 8.1
Ru-Ru	5.38 ± 0.85	2.656 ± 0.006	5.7 ± 0.9

Thus, from the above XAS analysis, we started from a black α-form hydrated $RuCl_3$ in water, which adopts the $CrCl_3$-type structure with long Ru-Ru interaction of 3.46 Å (As seen from the Fig. S12, the feature peak at the longer distance of 3.2 Å does not resemble to those features of RuO_2 standard. However, this matches more closer to the long-range Ru-Ru interaction (ca. 3.46 Å) retained in starting $RuCl_3$ species even after immobilization/hydrolysis). The Cl will be eventually replaced by the water/hydroxyl molecules during or after the immobilization. As seen from the EXAFS in the above figures after its immobilization, the peak at 1-2 Å (Ru-O) of X1.4 persists upon reduction and heat treatment (X1.4H and X1.4H-450) indicative of the Ru being surface bound by the surface oxygens. However, the decrease in coordination numbers in the nearest oxygens upon the treatments indicates the loss of mobile ligands

(anticipated to be H_2O/OH molecules) with the formation of a peak at 2-3 Å (Ru-Ru). This is believed to be the formation of small trapped Ru clusters in the 13X after the treatments. There is also no significant observable change for the peaks of X1.4H and X1.4H-450, showing that the nature of Ru sites does not change much even after the NH_3 decomposition. As a result, it is clear that from the above comparison, the octahedrally 6(O) surrounding the immobilized Ru in X1.4 will be dropped to ~3(O) with the formation of Ru-Ru bonding in the small Ru cluster trapped in the 13X. It is noted that we have recently reported a similar formation of small Ru clusters in HY zeolites (DOI: 10.1039/D2FD00175F Faraday Discuss., 2023, Advance Article). The Ru-Ru coordination number (CN) of 4-5 is much lower than those CN of Ru NPs on 6.4 wt.% Ru-Y sample.

H_2-TPR

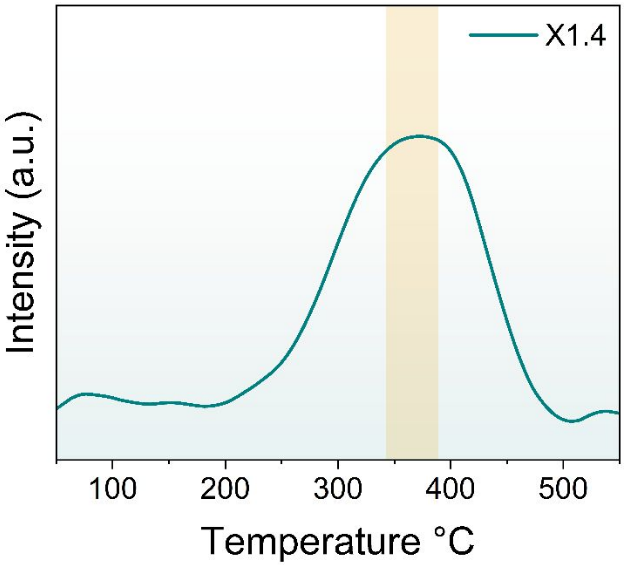

Figure S16. The H_2-TPR of the X1.4 sample.

N_2-TPD

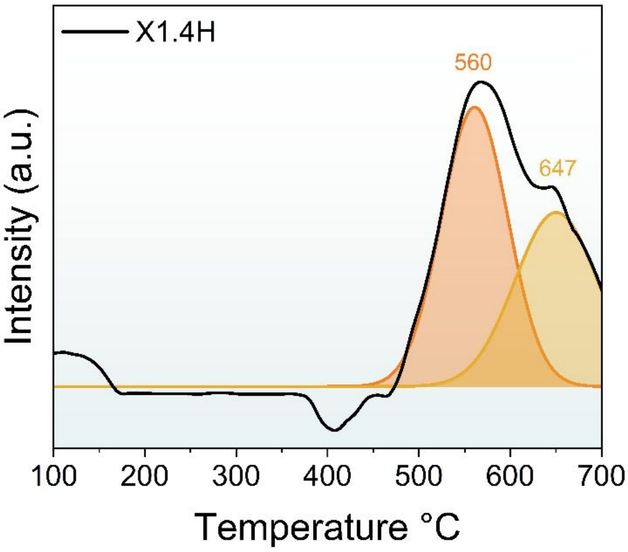

Figure S17. The N_2-TPD of the X1.4H sample (X1.4 sample with H_2 pre-treatment within the same instrument). The desorption of H_2 from BAS and Ru is taken place at about 400 ^oC (negative signal), followed by dehydroxylation process at higher temperatures.

BET surface area analysis

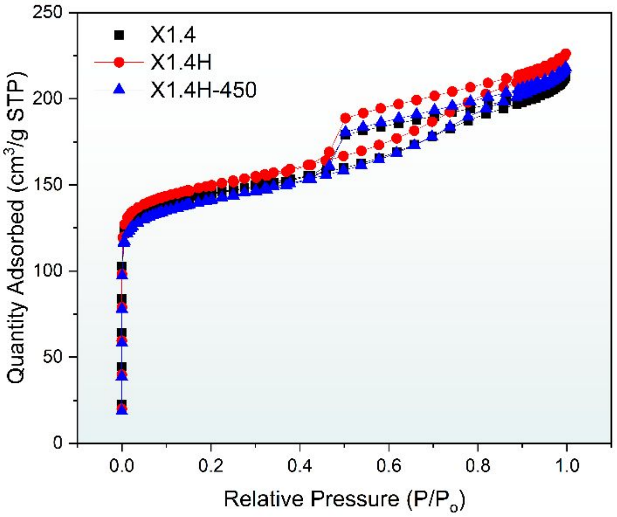

Figure S18. N_2 absorption-desorption isotherms of X1.4, X1.4H and X1.4H-450.

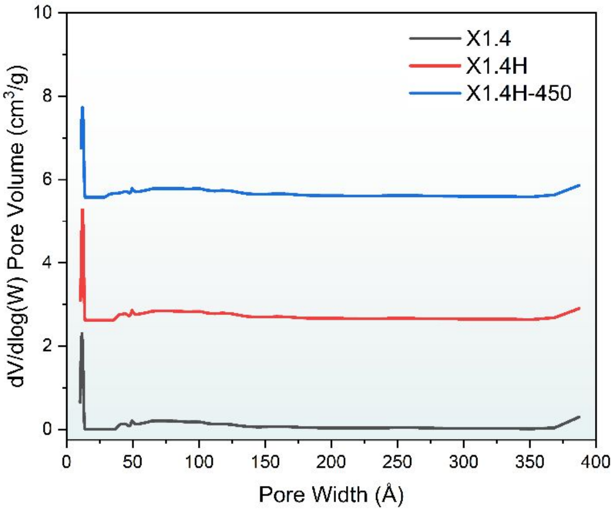

Figure S19. Pore size distributions of X1.4, X1.4H and X1.4H-450.

Table S5. Physicochemical characterization of X1.4, X1.4H and X1.4H-450.

	X1.4	X1.4H	X1.4H-450
BET Surface Area (m^2/g):	549.1756	566.4603	532.4336
t-Plot Micropore Area (m^2/g):	391.8222	403.8752	363.6250
t-Plot External Surface Area (m^2/g):	157.3534	162.5851	168.8086

Inductively coupled plasma mass spectrometry (ICP-MS)

Table S6. ICP-MS results of X0.25H, X0.4H, X0.8H, X1.4H, X3.0H and X4.8H with HF pre-treatment. 1% = 10,000,000 ppb.

Sample	Metal	Results (%)
X0.25H	Ru	0.25
X0.4H	Ru	0.4
X0.8H	Ru	0.8
X1.4H	Ru	1.4
X3.0H	Ru	3.0
X4.8H	Ru	4.8

TEM

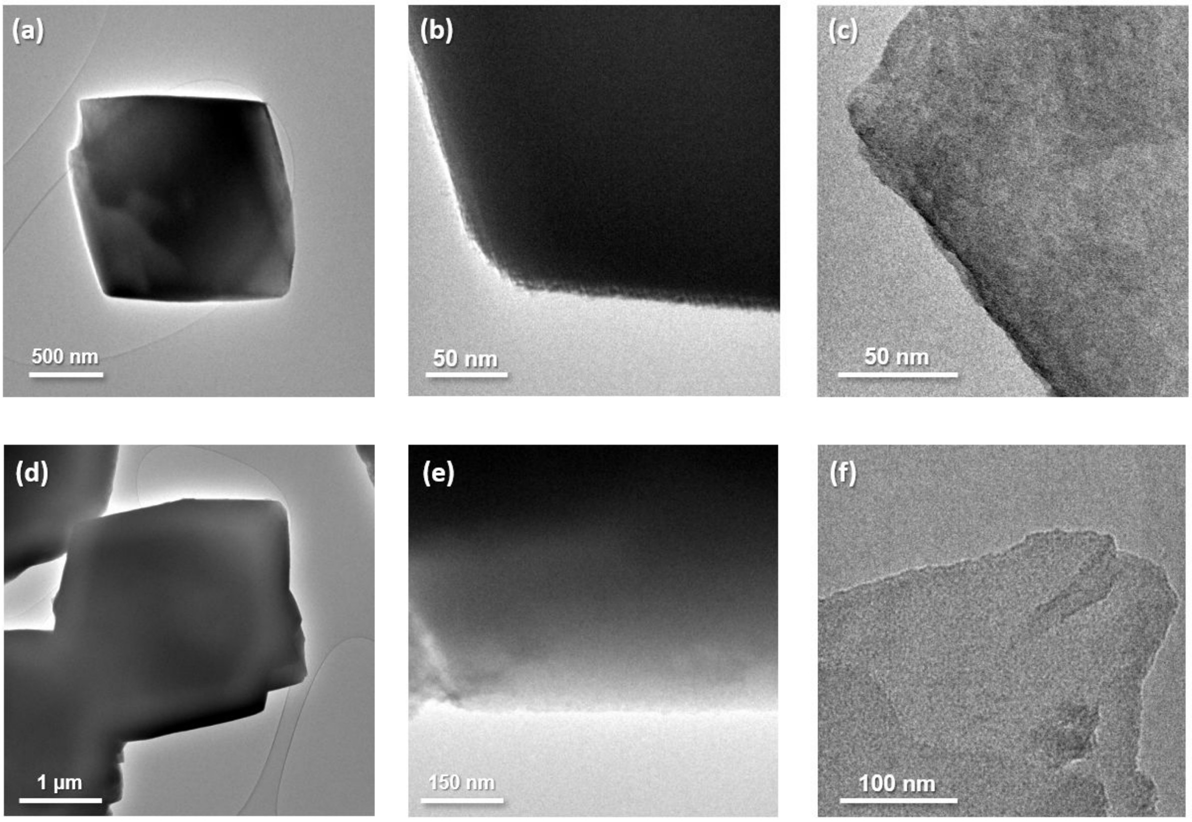

Figure S20. (a,b,c) The TEM images of X1.4. (d,e,f) The TEM images of X1.4H.

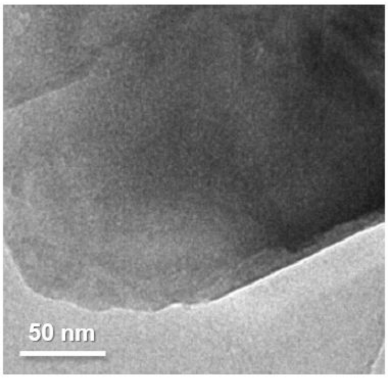

Figure S21. The TEM images of X1.4H-450.

***In-situ* mass spectrometry of X1.4H under NH_3/ND_3 decomposition**

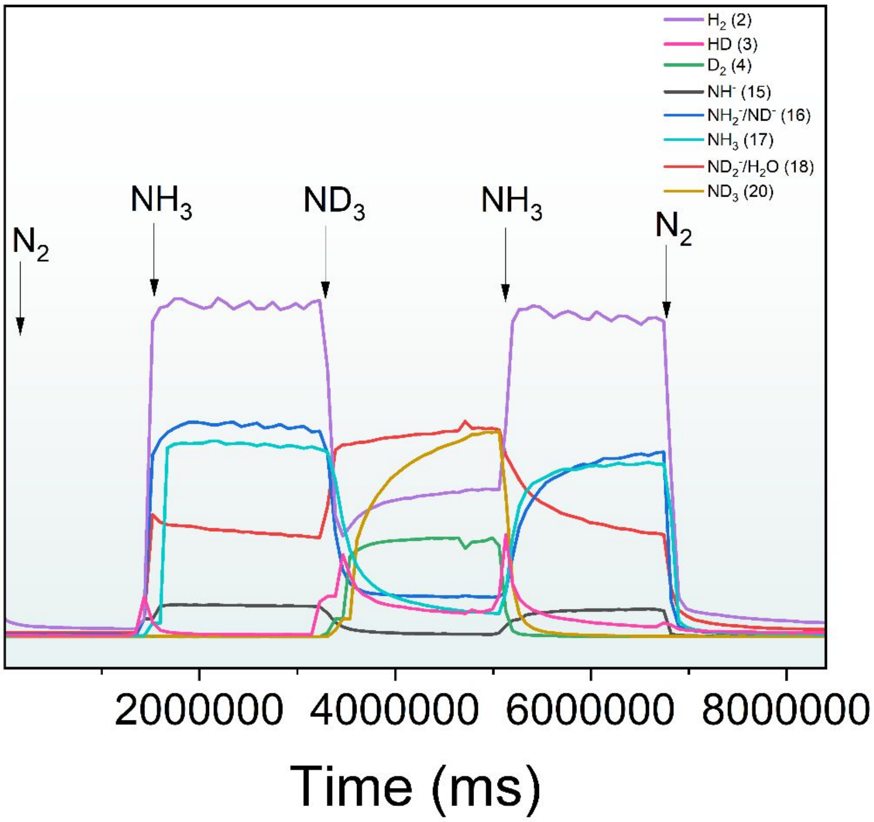

Figure S22. MID mode *in-situ* real time MS of X1.4H under NH_3/ND_3 decomposition at 450 °C. The gas flow was switched as the following order: N_2, NH_3, ND_3, NH_3 and N_2.

Stability test of X1.4H for Ammonia decomposition

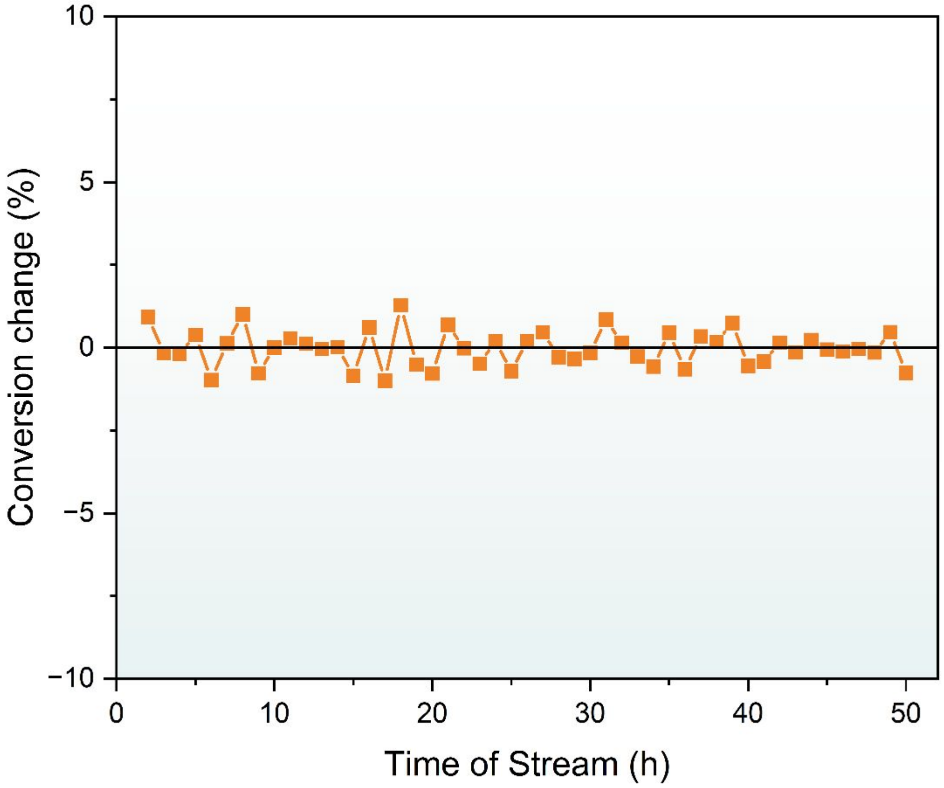

Figure S23. The conversion change (%) of the stability test of X1.4H for 50 hours of NH3 decomposition. The standard deviation of the conversion % is ± 0.60%.

Rietveld Refinement

Table S7. Crystallographic data and details of X1.4H based on SXRD.

Samples	X1.4H
Crystal system	Cubic
Space group	Fd-3z
2 theta range refinement	2°-50°
Detector	Multi-analyser crystals
Refinement methods	Rietveld
a (Å)	24.94(6)
b (Å)	24.94(6)
c (Å)	24.94(6)
V ($Å^3$)	15524.19(4)
Rwp/Rp/Rexp (%)	7.19/7.07/0.221
Wavelength	0.708721
2 theta zero point (°)	0
Gof χ^2	32.51

Table S8. Crystallographic information file from the Rietveld refinement of X1.4H based on SXRD.

Species	Atom	X	Y	z	SOF	Beq ($Å^2$)
Zeolite framework	Si1	-0.05149	0.03996	0.12059	1	0.14(02)
	Si2	-0.05438	0.12777	0.03301	1	0.14(02)
	O1	-0.10217	-0.00721	0.11002	1	0.28(05)
	O2	0.00051	-0.00070	0.14971	1	0.28(05)
	O3	-0.01931	0.07386	0.06737	1	0.28(05)
	O4	-0.06947	0.07939	0.17622	1	0.28(05)
Ru	Ru1	0.94072	0.44072	0.44072	0.08557	1(0)
	Ru2	0.98364	0.48364	0.26636	0.09564	2.18(94)
Na	Na1	0.06208	0.06208	0.06208	0.26589	4.96(74)
	Na2	0.23387	0.23387	0.23387	0.37470	1.38(26)

Table S9. Crystallographic data and details of X1.4H sample with NH_3 adsorption at room temperature (X1.4H-RT) and with NH_3 decomposition at 450 °C (X1.4H-450) based on NPD.

Samples	X1.4H-RT	X1.4H-450
Crystal system	Cubic	Cubic
Space group	Fd-3z	Fd-3z
Wavelength bands (Å)	2.45	2.45
Refinement methods	Rietveld	Rietveld
a (Å)	24.89	24.89
b (Å)	24.89	24.89
c (Å)	24.89	24.89
V (Å^3)	15425.49(6)	15425.49(6)
Rwp/Rp/Rexp (%)	2.38/1.87/1.87	2.00/1.52/1.50
Gof χ^2	1.27	1.33

Table S10. Crystallographic information file from the Rietveld refinement of X1.4H-RT based on NPD.

Species	Atom	X	Y	z	SOF	Beq (Å^2)
Zeolite framework	Si1	-0.05149	0.03996	0.12059	1	0.97(56)
	Si2	-0.05438	0.12777	0.03301	1	0.97(56)
	O1	-0.10217	-0.00721	0.11002	1	1.95(12)
	O2	0.00051	-0.00070	0.14971	1	1.95(12)
	O3	-0.01931	0.07386	0.06737	1	1.95(12)
	O4	-0.06947	0.07939	0.17622	1	1.95(12)
Ru	Ru1	0.94072	0.44072	0.44072	0.08557	1(0)
	Ru2	0.98364	0.48364	0.26636	0.09564	1(0)
Na	Na1	0.06208	0.06208	0.06208	0.26589	1(0)
	Na2	0.23387	0.23387	0.23387	0.37470	1(0)
H	H1	0.02300	0.09036	0.08027	0.19443	1(0)
NH_3 (near Ru1)	N1	0.89461	0.38772	0.47597	0.14111	1(0)
	H1a	0.89916	0.39026	0.51613	0.14111	1(0)
	H1b	0.85846	0.40378	0.46732	0.14111	1(0)
	H1c	0.89090	0.34764	0.47153	0.14111	1(0)
NH_3 (near Ru2)	N2	0.95904	0.43590	0.32606	0.22315	1(0)
	H2a	0.96669	0.47566	0.32655	0.22315	1(0)
	H2b	0.91958	0.43174	0.31797	0.22315	1(0)
	H2c	0.96387	0.42702	0.36527	0.22315	1(0)

Table S11. Crystallographic information file from the Rietveld refinement of X1.4H-450 based on NPD.

Species	Atom	X	Y	Z	SOF	Beq (Å^2)
Zeolite framework	Si1	-0.05149	0.03996	0.12059	1	0.97(56)
	Si2	-0.05438	0.12777	0.03301	1	0.97(56)
	O1	-0.10217	-0.00721	0.11002	1	1.95(12)
	O2	0.00051	-0.00070	0.14971	1	1.95(12)
	O3	-0.01931	0.07386	0.06737	1	1.95(12)
	O4	-0.06947	0.07939	0.17622	1	1.95(12)
Ru	Ru1	0.94072	0.44072	0.44072	0.08557	1(0)
	Ru2	0.98364	0.48364	0.26636	0.09564	1(0)
Na	Na1	0.06208	0.06208	0.06208	0.26589	1(0)
	Na2	0.23387	0.23387	0.23387	0.37470	1(0)
H	H1	0.01194	0.10446	0.07992	0.39590	2.28(54)
	H2	-0.00743	0.02447	0.18237	0.35578	1(0)
NH_3 (near Ru1)	N1	0.88514	0.39800	0.49020	0.13856	1(0)
	H1a	0.88969	0.40054	0.53036	0.13856	1(0)
	H1b	0.84899	0.41406	0.48155	0.13856	1(0)
	H1c	0.88143	0.35792	0.48576	0.13856	1(0)
NH_3 (near Ru2)	N2	0.95822	0.43807	0.32705	0.11336	1(0)
	H2a	0.96587	0.47783	0.32754	0.11336	1(0)
	H2b	0.91876	0.43391	0.31896	0.11336	1(0)
	H2c	0.96305	0.42919	0.36626	0.11336	1(0)

NPD-Rietveld refinement model

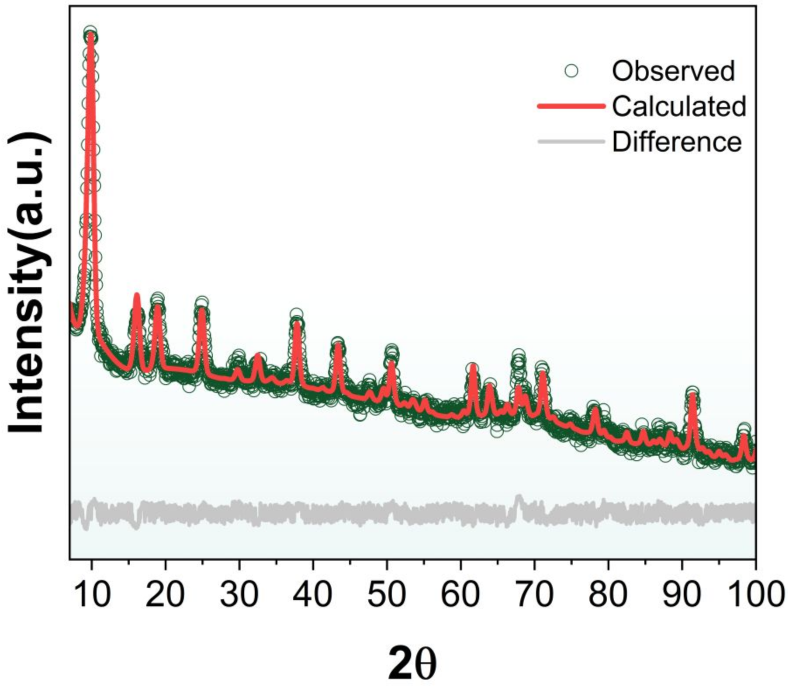

Figure S24. The Rietveld refinement profiles of NPD data of X1.4H-450 (R_{wp} = 1.998%, R_{exp} = 1.503%, gof = 1.3297).

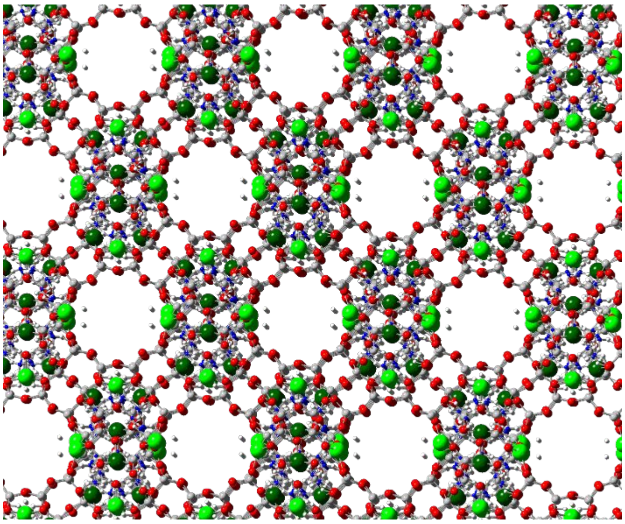

Figure S25. Structures of the determined proton sites and NH_3 molecules of X1.4H-450 via NPD-Rietveld refinement.

Bond length information of Rietveld refinement models

Table S12 Bond length information (in Å) of Rietveld refinement models.

	Ru1-O3	Ru2-O2	Ru1-Ru2	Ru1-N1	Ru2-N2	N1-N2
SXRD-refinement (X1.4H)	2.00(4)	2.17(3)	4.60(6)	/	/	/
NPD-refinement (X1.4H-RT)	2.00(0)	2.16(8)	4.59(6)	1.95(7)	1.99(9)	3.12(6)
NPD-refinement (X1.4H-450)	2.00(0)	2.16(8)	4.59(6)	2.13(6)	1.99(2)	3.09(2)

Computational

First N-H activation on the single-Ru catalyst

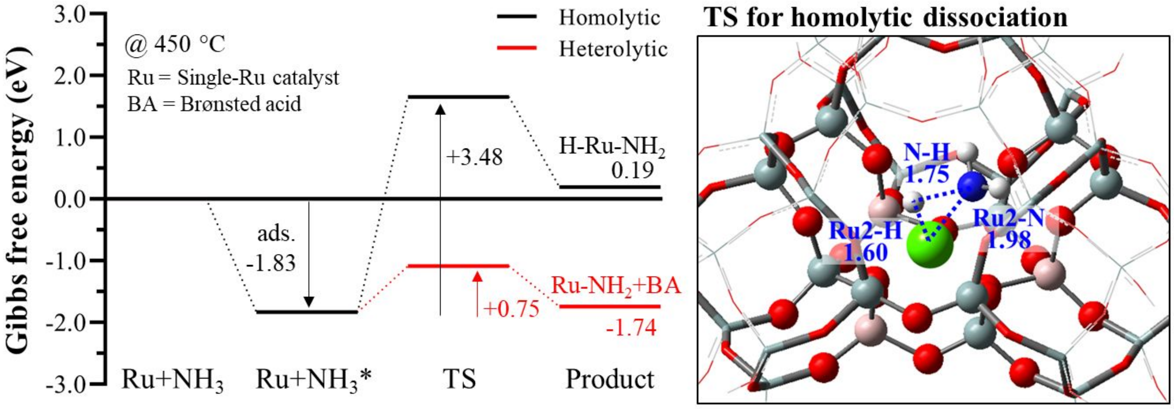

Figure S26. Gibbs free energy profile for homolytic and heterolytic N-H dissociation on the single-Ru(3+) catalyst determined at 450 °C (left). Optimized transition state structures for the homolytic N-H dissociation (right). Select interatomic distances are shown (in Å). The optimized transition state structure for the heterolytic dissociation is presented in Figure 2(b). Balls, tubes, and wires represent atoms in the high, middle, and low ONIOM layers, respectively. The atoms are colour coded according to element (green = Ru, red = O, grey = Si, pink = Al, and white = H).

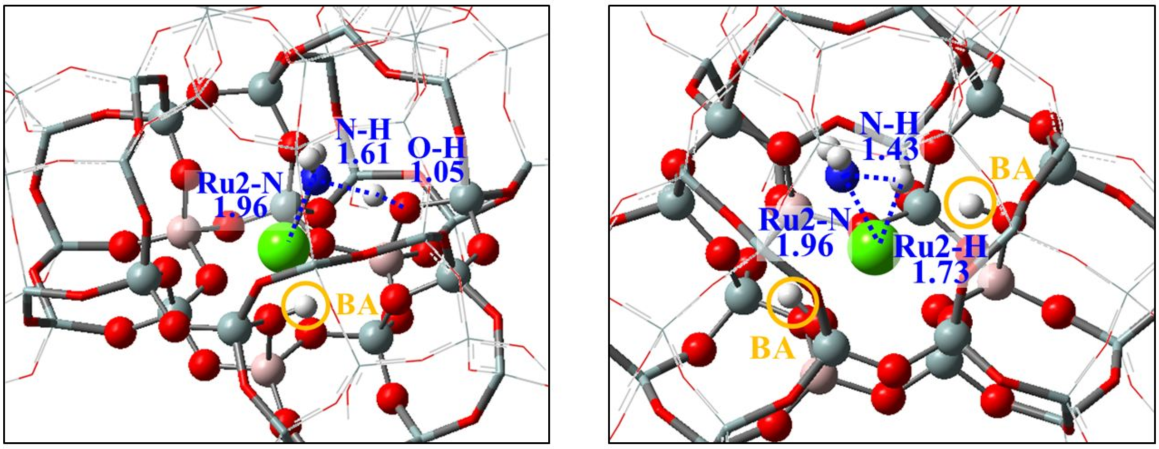

Figure S27. Optimized transition state structures for heterolytic N-H dissociation on Ru(2+) (left) and homolytic N-H dissociation on Ru(1+) (right). Select interatomic distances are shown (in Å).

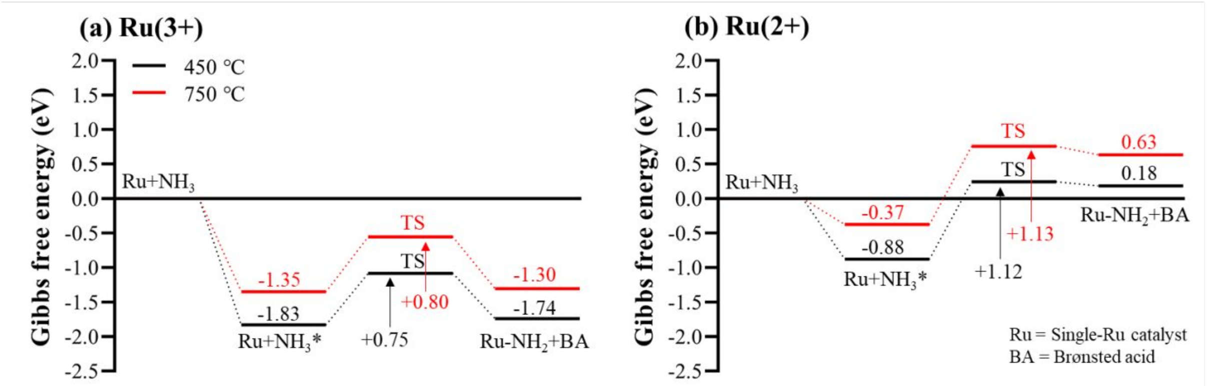

Figure S28. Gibb free energy profile for heterolytic N-H dissociation on the single-Ru catalysts with different oxidation states: (a) 3+ and (b) 2+, calculated at 450 °C (black) and 750 °C (red).

Full ammonia decomposition on the double-Ru catalyst

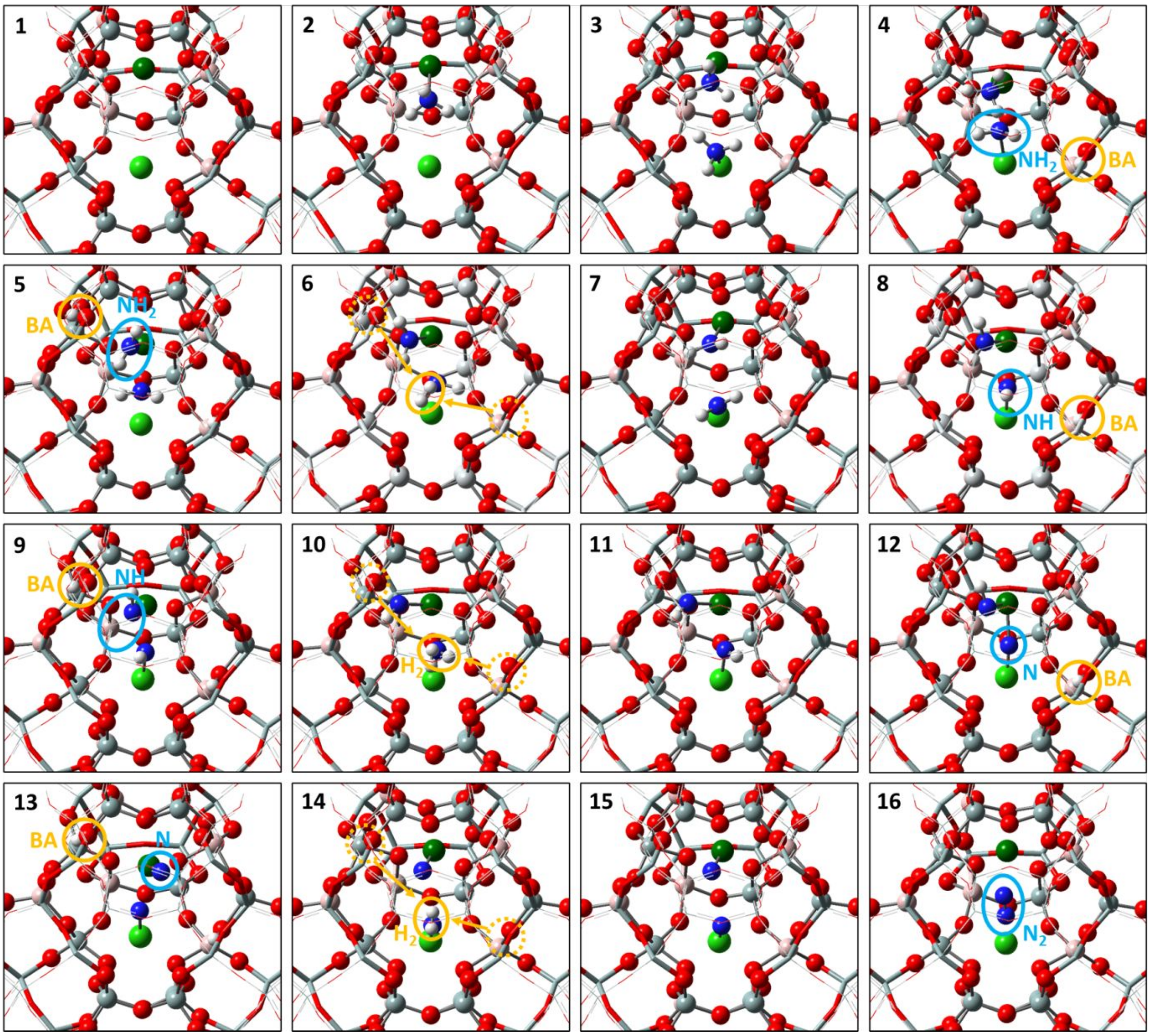

Figure S29. Optimized structures of each state in the full dissociation of two ammonia molecules ($2NH_3 \rightarrow N_2 + 3H_2$) on the double-Ru(3+) catalysts (Figure 8). The Brønsted acid sites, hydrogen molecules, NH_x (x=1 or 2) species, and nitrogen molecules involved in each reaction step of NH_3 decomposition are highlighted with circles.

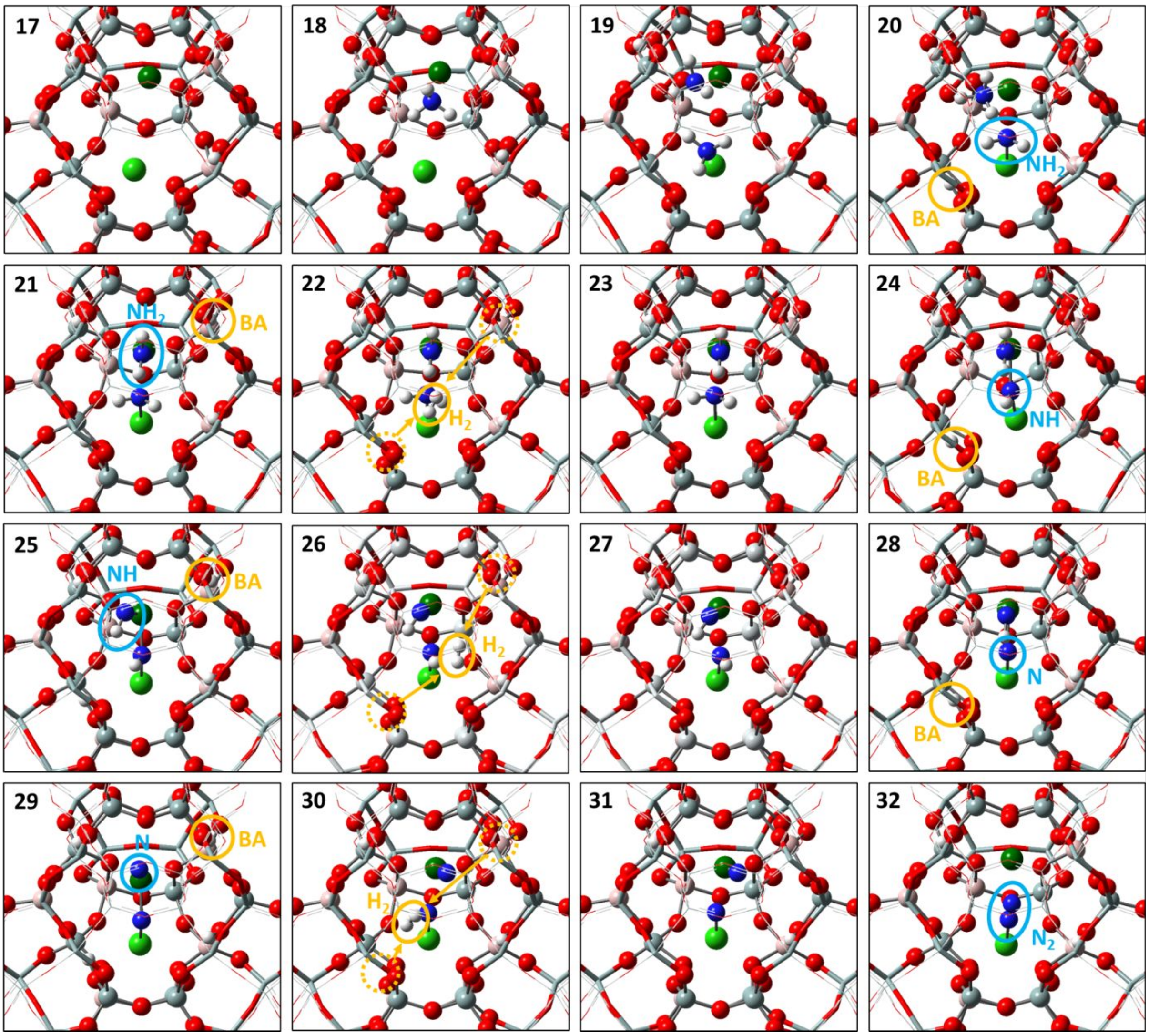

Figure S30. Optimized structures of each state in the full dissociation of two ammonia molecules ($2NH_3 \rightarrow N_2 + 3H_2$) on the double-Ru(2+) catalysts (Figure 8). The Brønsted acid sites, hydrogen molecules, NH_x (x=1 or 2) species, and nitrogen molecules involved in each reaction step of NH_3 decomposition are highlighted with circles.

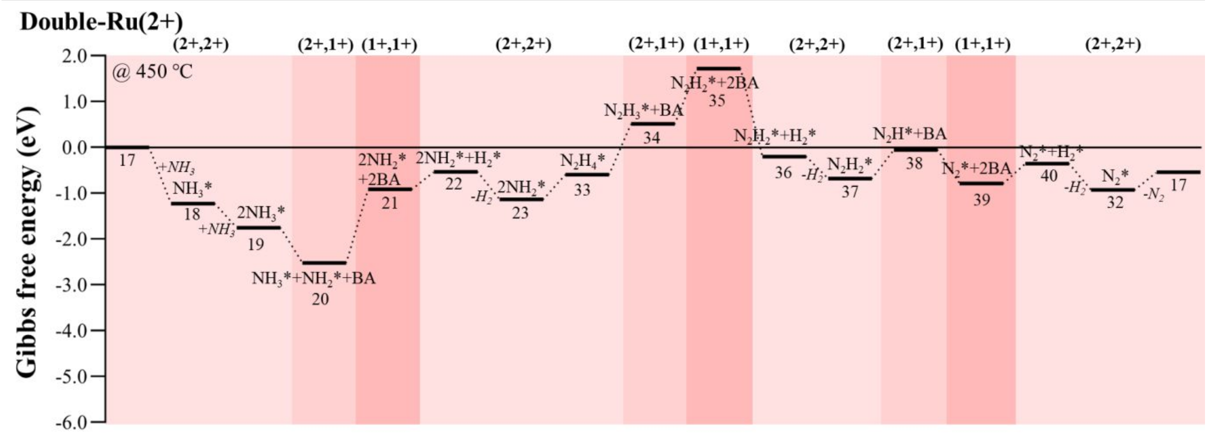

Figure S31. Gibbs free energy profile of full dissociation of two NH_3 molecules ($2NH_3 \rightarrow N_2 + 3H_2$) on double-Ru(2+) catalyst, involving formation of N_2H_4 intermediate. The formal oxidation states of the Ru atoms along the reaction coordinate are shown in parentheses. Asterisk (*) denotes adsorbed species on the catalysts.

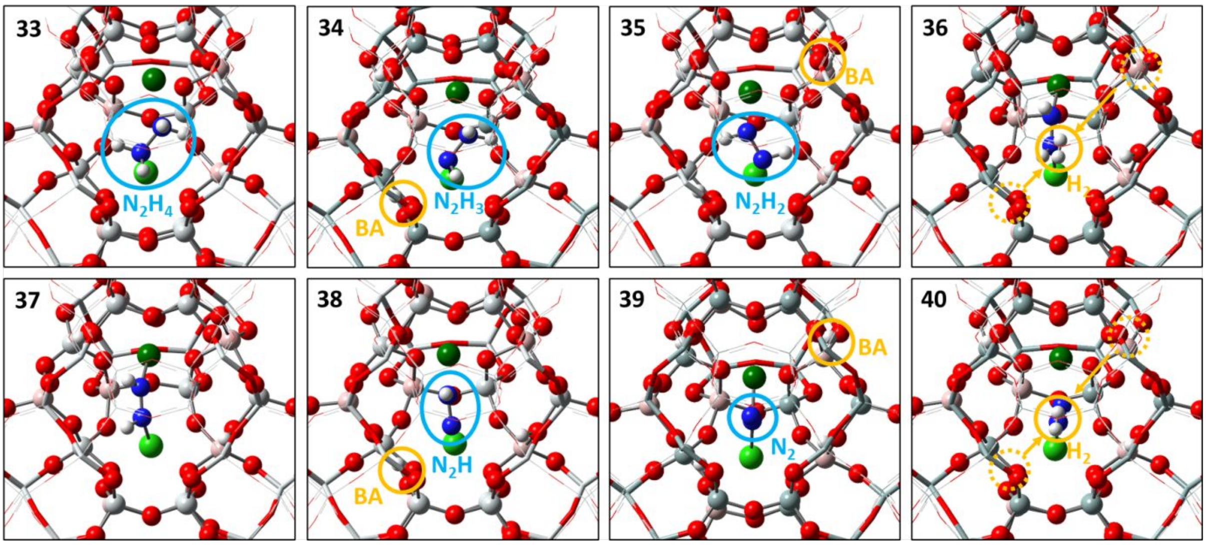

Figure S32. Optimized structures of each state in the full dissociation of two ammonia molecules ($2NH_3 \rightarrow N_2 + 3H_2$) on the double-Ru(2+) catalysts, following the N_2H_4-involved pathway (Figure S31). The Brønsted acid sites, hydrogen molecules, N_2H_x (x=2, 3, or 4) species, and nitrogen molecules involved in each reaction step of NH_3 decomposition are highlighted with circles. Repeated structures are omitted.

Bond length information from the computational chemistry models

Double-Ru(3+), without NH_3

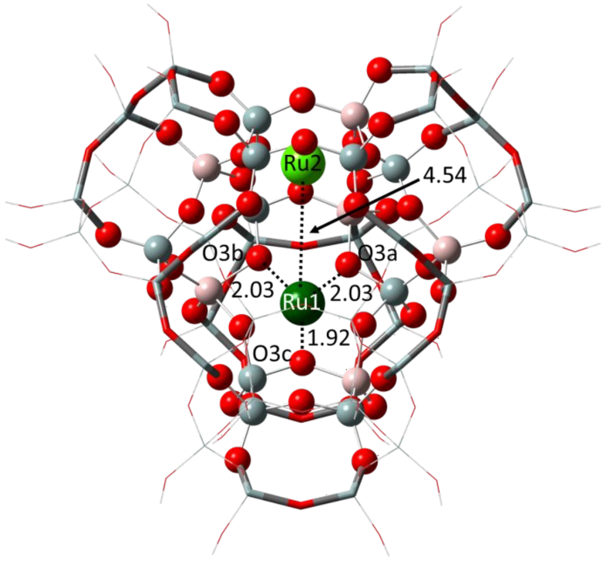

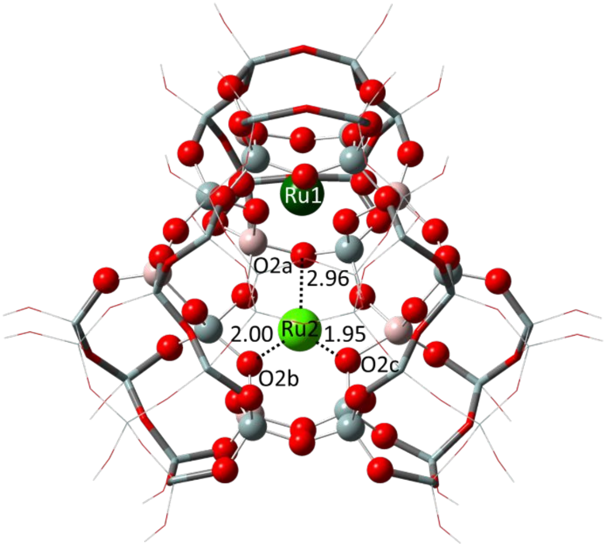

	Ru1-O3	Ru2-O2	Ru-Ru
Average distance (Å)	1.99	2.31	4.54
Standard deviation	0.06	0.57	

Figure S33. Bond length information (in Å) of the optimized Double-Ru(3+) catalyst without NH_3.

Double-Ru(3+), with two NH_3

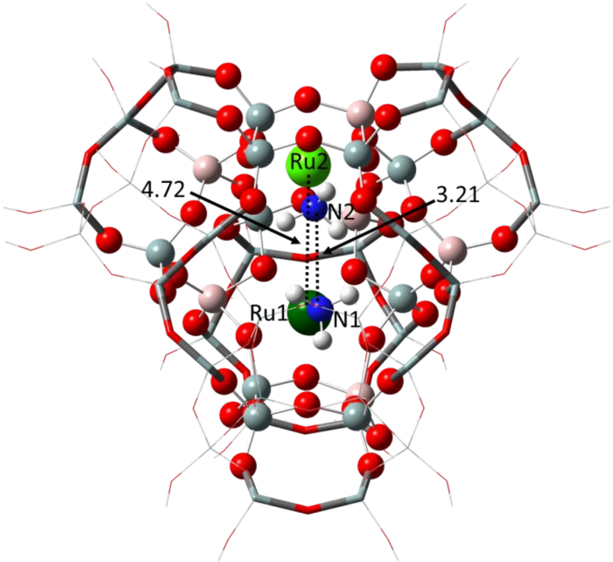

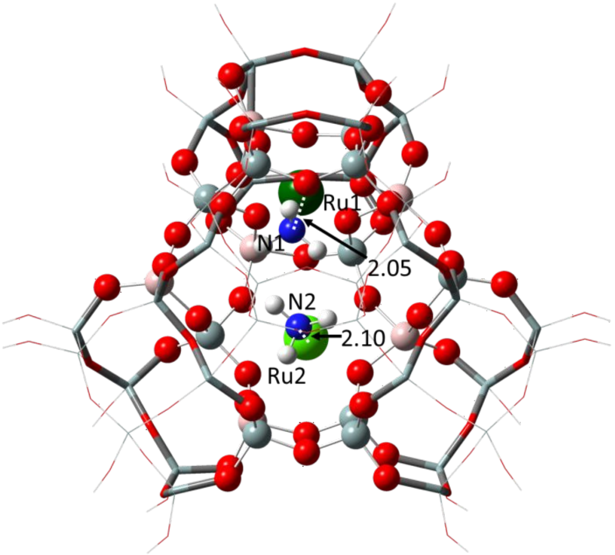

	Ru1-O3	Ru2-O2	Ru-Ru	Ru1-N1	Ru2-N2	N1-N2
Average distance (Å)	2.02	2.43	4.72	2.05	2.10	3.21
Standard deviation	0.03	0.55				

Figure S34. Bond length information (in Å) of the optimized Double-Ru(3+) catalyst with two NH_3 molecules adsorbed on each Ru centre.

Double-Ru(2+), without NH_3

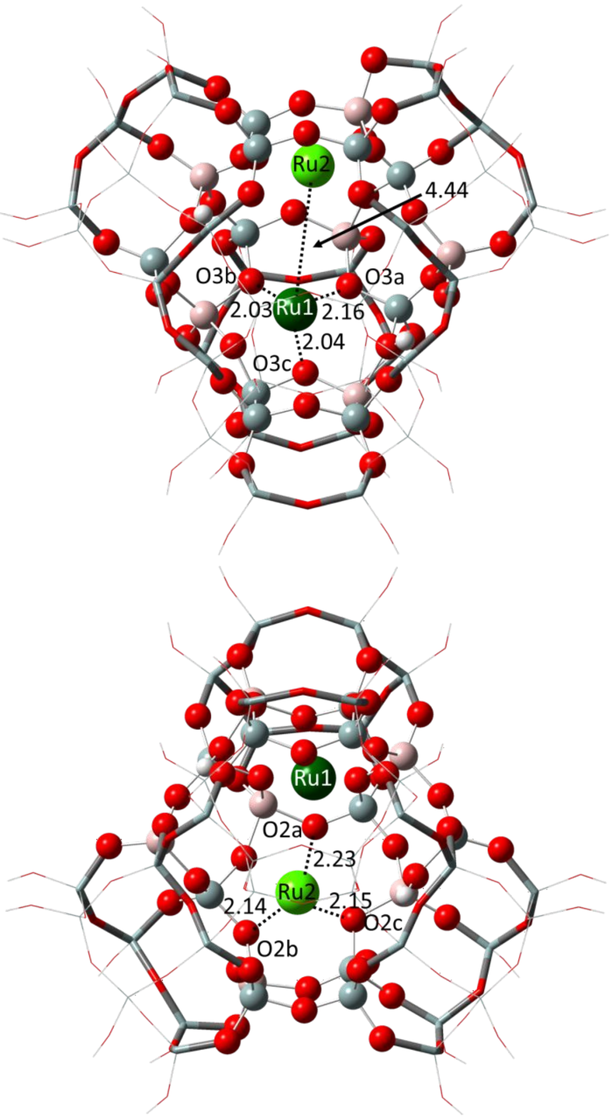

	Ru1-O3	Ru2-O2	Ru-Ru
Average distance (Å)	2.07	2.17	4.44
Standard deviation	0.07	0.05	

Figure S35. Bond length information (in Å) of the optimized Double-Ru(2+) catalyst without NH_3.

Double-Ru(2+), with two NH_3

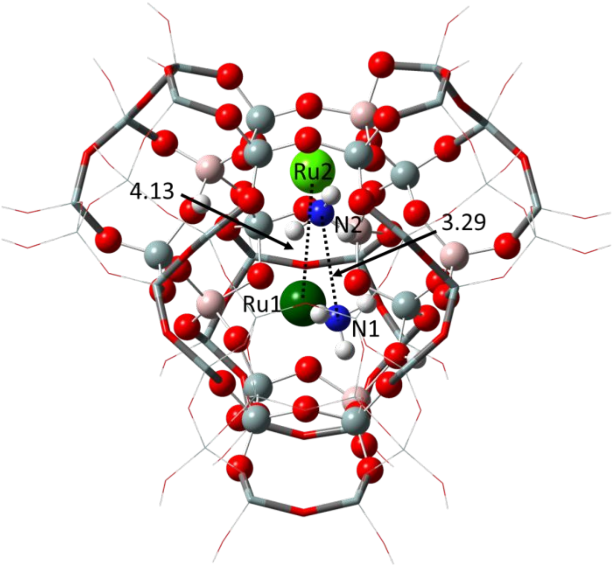

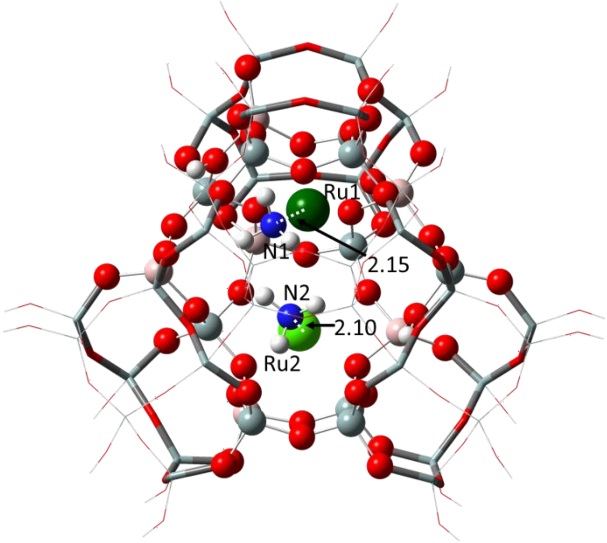

	Ru1-O3	Ru2-O2	Ru-Ru	Ru1-N1	Ru2-N2	N1-N2
Average distance (Å)	2.13	2.43	4.13	2.15	2.10	3.29
Standard deviation	0.02	0.45				

Figure S36. Bond length information (in Å) of the optimized Double-Ru(2+) catalyst with two NH_3 molecules adsorbed on each Ru centre.

Single-Ru(3+) (top) and single Ru(2+) (bottom), without NH_3

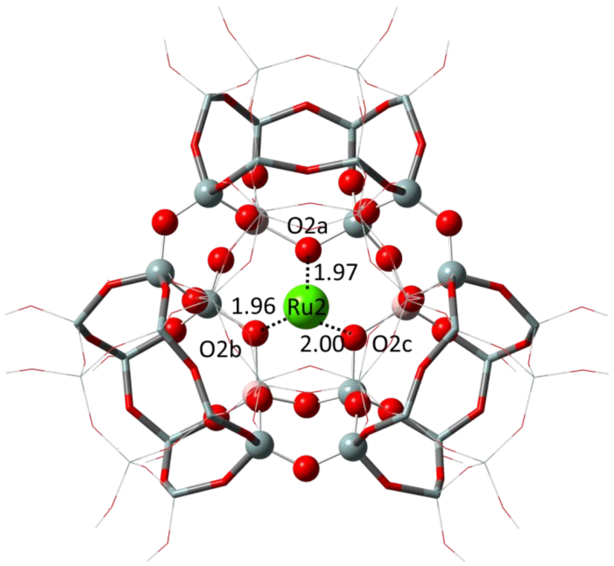

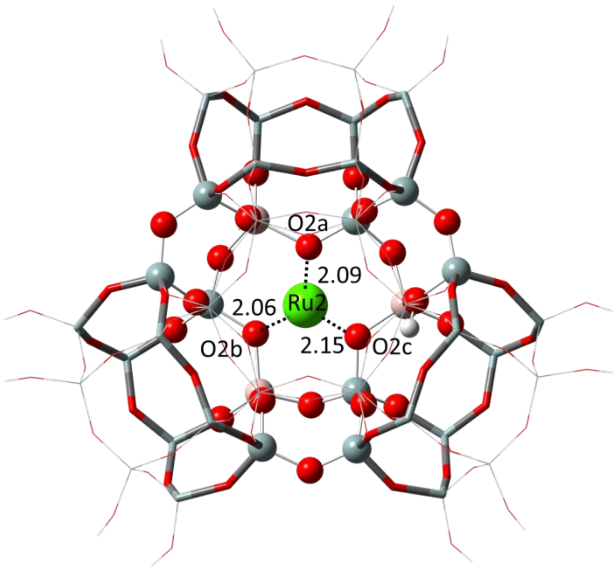

	Ru2-O2 (Ru(3+))	Ru2-O2 (Ru(2+))
Average distance (Å)	1.98	2.10
Standard deviation	0.02	0.05

Figure S37. Bond length information (in Å) of the optimized Single-Ru(3+) (top) and Single-Ru(2+) (bottom) catalysts without NH_3.

Reference

1. Dapprich, S.; Komáromi, I.; Byun, K. S.; Morokuma, K.; Frisch, M. J., A new ONIOM implementation in Gaussian98. Part I. The calculation of energies, gradients, vibrational frequencies and electric field derivatives. *Journal of Molecular Structure: THEOCHEM* **1999,** *461*, 1-21.
2. Humbel, S.; Sieber, S.; Morokuma, K., The IMOMO method: Integration of different levels of molecular orbital approximations for geometry optimization of large systems: Test for n-butane conformation and SN 2 reaction: RCl+ Cl−. *The Journal of chemical physics* **1996,** *105* (5), 1959-1967.
3. Svensson, M.; Humbel, S.; Froese, R. D. J.; Matsubara, T.; Sieber, S.; Morokuma, K., ONIOM: a multilayered integrated MO+ MM method for geometry optimizations and single point energy predictions. A test for Diels− Alder reactions and Pt (P (t-Bu) 3) 2+ H2 oxidative addition. *The Journal of Physical Chemistry* **1996,** *100* (50), 19357-19363.
4. Vreven, T.; Morokuma, K., On the application of the IMOMO (integrated molecular orbital+ molecular orbital) method. *Journal of Computational Chemistry* **2000,** *21* (16), 1419-1432.
5. Frisch, M. J.; Trucks, G. W.; Schlegel, H. B.; Scuseria, G. E.; Robb, M. A.; Cheeseman, J. R.; Scalmani, G.; Barone, V.; Mennucci, B.; Petersson, G. A., Gaussian 09, Revision D. 01, Gaussian, Inc., Wallingford CT. **2009**.
6. Namuangruk, S.; Pantu, P.; Limtrakul, J., Alkylation of benzene with ethylene over faujasite zeolite investigated by the ONIOM method. *Journal of Catalysis* **2004,** *225* (2), 523-530.
7. Shiota, Y.; Suzuki, K.; Yoshizawa, K., QM/MM study on the catalytic mechanism of benzene hydroxylation over Fe− ZSM-5. *Organometallics* **2006,** *25* (13), 3118-3123.
8. Gomes, J.; Zimmerman, P. M.; Head-Gordon, M.; Bell, A. T., Accurate prediction of hydrocarbon interactions with zeolites utilizing improved exchange-correlation functionals and QM/MM methods: benchmark calculations of adsorption enthalpies and application to ethene methylation by methanol. *The Journal of Physical Chemistry C* **2012,** *116* (29), 15406-15414.
9. Kostetskyy, P.; Mpourmpakis, G., Computational insights into adsorption of C4 hydrocarbons in cation-exchanged ZSM-12 zeolites. *Industrial & Engineering Chemistry Research* **2017,** *56* (24), 7062-7069.
10. Chai, J.-D.; Head-Gordon, M., Long-range corrected hybrid density functionals with damped atom–atom dispersion corrections. *Physical Chemistry Chemical Physics* **2008,** *10* (44), 6615-6620.
11. Stewart, J. J. P., Optimization of parameters for semiempirical methods V: Modification of NDDO approximations and application to 70 elements. *Journal of Molecular modeling* **2007,** *13* (12), 1173-1213.
12. Rappé, A. K.; Casewit, C. J.; Colwell, K.; Goddard III, W. A.; Skiff, W. M., UFF, a full periodic table force field for molecular mechanics and molecular dynamics simulations. *Journal of the American chemical society* **1992,** *114* (25), 10024-10035.
13. Hay, P. J.; Wadt, W. R., Ab initio effective core potentials for molecular calculations. Potentials for the transition metal atoms Sc to Hg. *The Journal of chemical physics* **1985,** *82* (1), 270-283.
14. Hay, P. J.; Wadt, W. R., Ab initio effective core potentials for molecular calculations. Potentials for K to Au including the outermost core orbitals. *The Journal of chemical physics* **1985,** *82* (1), 299-310.
15. Wadt, W. R.; Hay, P. J., Ab initio effective core potentials for molecular calculations. Potentials for main group elements Na to Bi. *The Journal of chemical physics* **1985,** *82* (1), 284-298.
16. Frisch, A. E.; Foresman, J. B., *Exploring chemistry with electronic structure methods*. 3rd ed.; Gaussian Inc: Wallingford, CT, USA, 2015.
17. Cha, J.; Lee, T.; Lee, Y.-J.; Jeong, H.; Jo, Y. S.; Kim, Y.; Nam, S. W.; Han, J.; Lee, K. B.; Yoon, C. W.; Sohn, H., Highly monodisperse sub-nanometer and nanometer Ru particles confined in alkali-exchanged zeolite Y for ammonia decomposition. *Applied Catalysis B: Environmental* **2021,** *283*.
18. Manabe, R.; Nakatsubo, H.; Gondo, A.; Murakami, K.; Ogo, S.; Tsuneki, H.; Ikeda, M.; Ishikawa, A.; Nakai, H.; Sekine, Y., Electrocatalytic synthesis of ammonia by surface proton hopping. *Chem Sci* **2017,** *8* (8), 5434-5439.

19. Yu, C.; Huang, B.; Dong, L.; Chen, F.; Liu, X., In situ FT-IR study of highly dispersed MnO x /SAPO-34 catalyst for low-temperature selective catalytic reduction of NO x by NH 3. *Catalysis Today* **2017,** *281*, 610-620.
20. Kijlstra, W. S.; Brands, D. S.; Smit, H. I.; Poels, E. K.; Bliek, A., Mechanism of the Selective Catalytic Reduction of NO with NH3over MnOx/Al2O3. *Journal of Catalysis* **1997,** *171* (1), 219-230.
21. Boroń, P.; Chmielarz, L.; Gil, B.; Marszałek, B.; Dzwigaj, S., Experimental evidence of NO SCR mechanism in the presence of the BEA zeolite with framework and extra-framework cobalt species. *Applied Catalysis B: Environmental* **2016,** *198*, 457-470.
22. Osuga, R.; Yokoi, T.; Kondo, J. N., IR observation of activated ether species on acidic OH groups on H-ZSM-5 zeolites. *Molecular Catalysis* **2019,** *477*.
